# A Pharmacometric Analysis of Patient-Reported Outcomes in Breast Cancer Patients Through Item Response Theory

**DOI:** 10.1007/s11095-018-2403-8

**Published:** 2018-04-19

**Authors:** Emilie Schindler, Lena E. Friberg, Bertram L. Lum, Bei Wang, Angelica Quartino, Chunze Li, Sandhya Girish, Jin Y. Jin, Mats O. Karlsson

**Affiliations:** 1 Department of Pharmaceutical Biosciences, Uppsala University, Box 591, SE-75124 Uppsala, Sweden; 2 Department of Clinical Pharmacology, Genentech Inc., South San Francisco, California, USA

**Keywords:** ado-trastuzumab emtansine, capecitabine, kadcyla, lapatinib, nonlinear mixed effects, NONMEM, T-DM1

## Abstract

**Purpose:**

An item response theory (IRT) pharmacometric framework is presented to characterize Functional Assessment of Cancer Therapy-Breast (FACT-B) data in locally-advanced or metastatic breast cancer patients treated with ado-trastuzumab emtansine (T-DM1) or capecitabine-plus-lapatinib.

**Methods:**

In the IRT model, four latent well-being variables, based on FACT-B general subscales, were used to describe the physical, social/family, emotional and functional well-being. Each breast cancer subscale item was reassigned to one of the other subscales. Longitudinal changes in FACT-B responses and covariate effects were investigated.

**Results:**

The IRT model could describe both item-level and subscale-level FACT-B data. Non-Asian patients showed better baseline social/family and functional well-being than Asian patients. Moreover, patients with Eastern Cooperative Oncology Group performance status of 0 had better baseline physical and functional well-being. Well-being was described as initially increasing or decreasing before reaching a steady-state, which varied substantially between patients and subscales. T-DM1 exposure was not related to any of the latent variables. Physical well-being worsening was identified in capecitabine-plus-lapatinib-treated patients, whereas T-DM1-treated patients typically stayed stable.

**Conclusion:**

The developed framework provides a thorough description of FACT-B longitudinal data. It acknowledges the multi-dimensional nature of the questionnaire and allows covariate and exposure effects to be evaluated on responses.

**Electronic supplementary material:**

The online version of this article (10.1007/s11095-018-2403-8) contains supplementary material, which is available to authorized users.

## Introduction

In the era of targeted therapies, metastatic cancers are becoming chronic-like diseases, for which a goal of treatments is to maintain functioning and improve quality of life. While traditional outcome measures focusing on overall survival and progression-free survival are essential in cancer decision making, there has been growing evidence that patient-reported outcome (PRO) measures convey additional information for assessing the overall burden of cancer, tolerability, and effectiveness of interventions over long treatment durations, where applicable (1,2). PRO measures are reports about how a patient feels and functions in relation to a disease and its treatment, that come directly from the patient without interpretation of the response by a clinician or any third party (3). PRO measures are usually collected through questionnaires consisting of several items (questions). While some PRO measures focus on single aspects of health-related quality of life (HRQoL) (e.g. the diarrhoea assessment scale), others are designed to evaluate multiple elements of HRQoL (e.g. the European Organisation for Research and Treatment of Cancer Quality of Life Questionnaire (EORTC QLQ-C30), the Functional Assessment of Cancer Therapy-General (FACT-G)), including disease symptoms, drug-related toxicities, physical functioning, and social and psychological well-being. These aspects of HRQoL are important for multiple stakeholders, including sponsors, regulatory agencies, payers, prescribers and patients. Interestingly, patients have been shown to report symptoms earlier and more frequently than clinicians. However, clinician reports of fatigue, nausea, constipation, and performance status were associated with death and emergency room admissions, whereas no association was identified when patients reported those items. Moreover, patient-reported symptoms were more often in agreement with measures of daily health status than clinician-reported symptoms (4,5). Multi-item PRO questionnaires are therefore likely to provide a comprehensive picture of a patient’s well-being.

Multi-item PRO data are traditionally analysed by calculating the sum of the item scores, and criteria based on expert opinion, such as time to symptom worsening, are then derived. Despite quick computing and easy interpretation, the use of these composite scores results in a loss of information (at both longitudinal and item level) and involves technical challenges, including missing data imputation. Alternatively, item-response theory (IRT), used extensively in educational testing applications, has gained in popularity and acceptance in PRO research. IRT can help address these practical problems and provide richer and more accurate description of item-level PRO questionnaire data (6,7). IRT consists of a statistical framework in which a set of mathematical models are used to describe the relationship between patients’ “latent” (i.e. unobservable) status and how they respond to items. This relationship can be visualized on item characteristic curves (ICCs). IRT models have recently been applied in a pharmacometric framework (8) to various disease areas such as Alzheimer’s disease (9), multiple sclerosis (10), schizophrenia (11), Parkinson’s disease (12,13) and cognition in the elderly (14). IRT pharmacometric models were shown to have an increased statistical power to detect drug effect when compared to composite scores (9,13).

Ado-trastuzumab emtansine (T-DM1), an antibody-drug conjugate that combines the antitumor properties of the human epidermal growth factor receptor 2 (HER2)-targeted antibody trastuzumab with the cytotoxic activity of the microtubule inhibitor emtansine (DM1), was granted regulatory approval in the United States, Europe and elsewhere for the treatment of HER2-positive metastatic breast cancer previously treated with trastuzumab and taxane chemotherapy (15). Approval was based on the results from the phase III EMILIA trial, which demonstrated a better safety profile and an improved progression-free and overall survival in HER2-positive locally advanced or metastatic breast cancer patients treated with T-DM1 when compared to lapatinib plus capecitabine, a standard of care chemotherapy-based treatment (16). Previous findings from exposure-response analyses of EMILIA overall survival and progression-free survival data have suggested that there may be an opportunity to optimize the dose in the subgroup of patients displaying low T-DM1 exposure for improved efficacy with acceptable tolerability (17).

PRO data are increasingly being implemented in oncology clinical trial research, and we therefore aimed to develop of a methodology that combines IRT and pharmacometric approaches to describe longitudinal PRO data in cancer patients, using FACT-Breast (FACT-B) data from EMILIA trial. Specific aims were to characterize the time-course of item-level FACT-B questionnaire data using IRT methods following treatment with T-DM1, to investigate potential exposure-response relationships, and to compare the PRO responses to T-DM1 and capecitabine-plus-lapatinib treatment for locally advanced/metastatic breast cancer. Methodological aspects for the development and implementation of the model, as well as parameters defining the ICCs, are presented.

## Methods

### Study Design

**Fig. 1:**
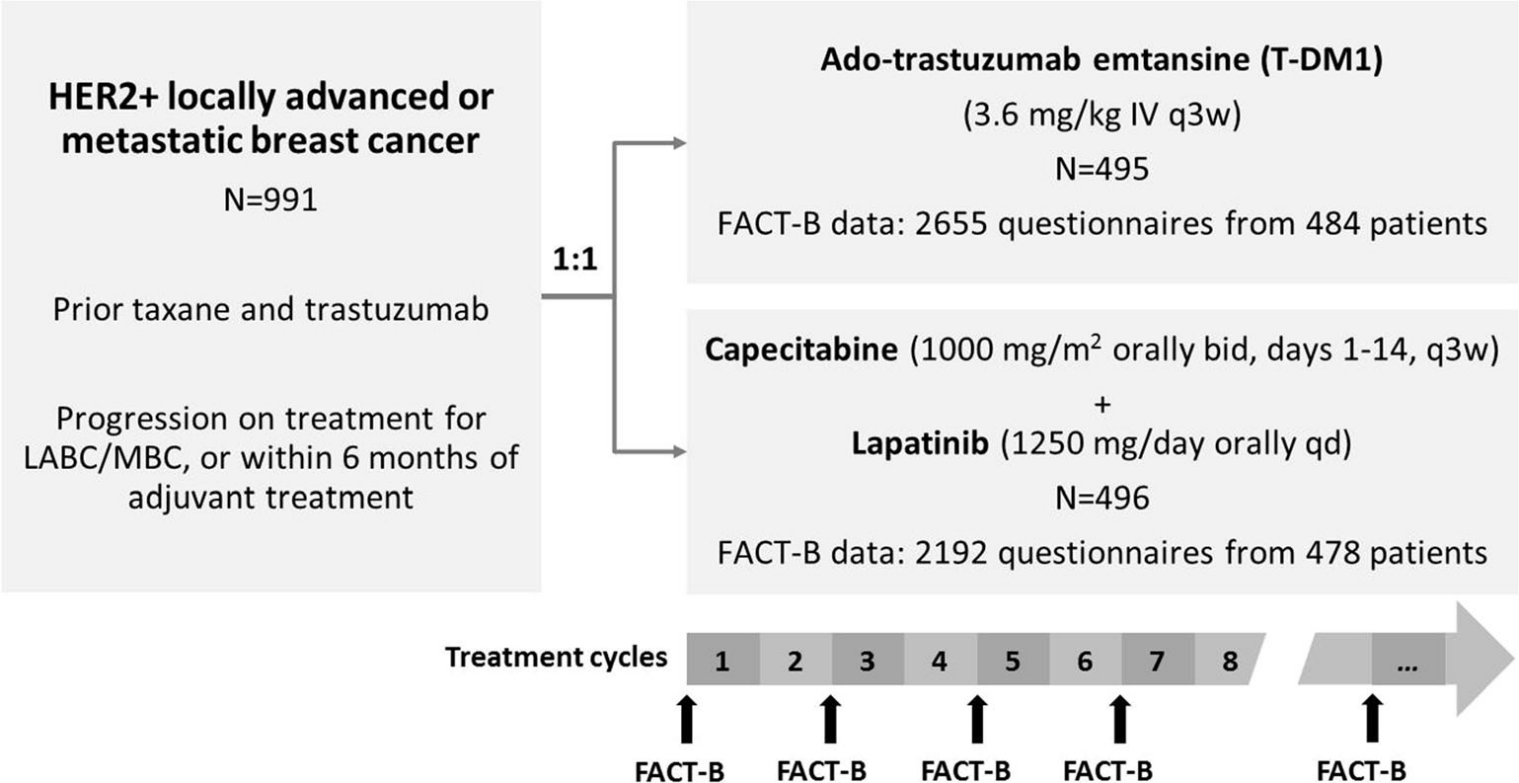
Overview of EMILIA study design and functional assessment of cancer therapy-breast (FACT-B) data considered in the modeling analysis. HER2+: human epidermal growth factor receptor 2-positive; N: number of patients randomized; LABC: locally advanced breast cancer; MBC: metastatic breast cancer; IV: intravenous; q3w: every three weeks; bid: twice daily; qd: once daily. Figure adapted from Welslau *et al.*

Data from the EMILIA clinical trial (NTC00829166) were included in this analysis. EMILIA was a randomized, open-label, international pivotal phase III study, involving patients with HER2-positive, unresectable locally advanced or metastatic breast cancer, previously treated with trastuzumab and a taxane (16). A total of 991 patients were randomized in a 1:1 ratio to T-DM1 (3.6 mg/kg intravenously every three weeks (q3w)) or capecitabine-plus-lapatinib (active control treatment) until disease progression or unmanageable toxicity (Fig. 1). Both treatments were given in three-week cycles. The EMILIA study was conducted in accordance with the Declaration of Helsinki and Good Clinical Practice guidelines. Written informed consent was obtained from all patients and with the relevant institutional review board and/or independent ethics committee at each study site approving the study protocol.

### FACT-B and Pharmacokinetic Assessments

The FACT-B questionnaire (version 4) consists of 36 items with ordered categorical answers. Possible responses to each item are “Not at all”, “A little bit”, “Somewhat”, “Quite a bit” and “Very much”. As per FACT-B scoring guidelines (http://www.facit.org/FACITOrg/Questionnaires), for non-reversed items (e.g. “I feel close to my friends”, “I am sleeping well”) these answers correspond to scores of 0, 1, 2, 3 and 4, respectively, whereas for reverse items (e.g. “I have nausea”, “I feel sad”) they correspond to scores of 4, 3, 2, 1 and 0, respectively (i.e. the higher the score the better the quality of life). A list of all FACT-B items is provided in ***Supplementary Table*** ***S1***. FACT-B items are grouped into five subscales: the physical, social/family, emotional and functional well-being subscales, which form the FACT-General scale, and an additional breast cancer-specific subscale (BCS). Item-level FACT-B data considered in this modeling analysis were collected before the start of treatment (baseline, day 1 of cycle 1) and on day 1 of every odd cycle (i.e. every 6 weeks), and excluded the study completion visit (Fig. 1). A total of 2655 FACT-B responses were available from 484 female patients in the T-DM1 arm (out of 495 patients randomized to this arm), with a median follow-up duration of 24 weeks (range, 0–117). In the capecitabine-plus-lapatinib arm, 2192 questionnaire data were collected from 478 female patients followed up for a median duration of 18 weeks (range, 0–130). The percentage of missing answers ranged from 0.1–4% for all items, except for one item (“Sex life”) with 43% of missing data.

Pharmacokinetic data were available for a subset of 331 patients in the T-DM1 treated group. Cycle 1 area under the concentration-time curve (AUC_cycle1_) and cycle 1 minimum concentration (C_min,cycle1_, at a nominal time of 504 h) were obtained from a published population pharmacokinetic model (18). For patients on T-DM1 missing pharmacokinetic data (*N* = 153), AUC_cycle1_ and C_min,cycle1_ were calculated based on the typical pharmacokinetic parameters corrected for individual covariates.

### IRT and Longitudinal Well-Being Modeling

The IRT model was developed in three steps, which included: 1) a base IRT model, where the probability of each item score was linked to a latent variable, 2) a longitudinal well-being model, where the trajectory of the latent variable was modelled as a function of explanatory variables (time, covariates), and 3) a longitudinal IRT model, where models from the two previous steps were combined to describe longitudinal changes in the probability of each item score, including potential covariate effects. Each step is detailed below.

#### Base IRT Model

The model was developed using T-DM1 arm data. It was then evaluated on capecitabine-plus-lapatinib arm data.

The IRT model describes the probability of each score for any given item as a function of a patient’s well-being (*W*), which is a latent variable. Since each FACT-B subscale relates to a specific aspect of well-being, the IRT model included several latent variables (*W*_*i*, *l*_ specific to patient *i* and subscale *l*). Each item was modeled using a proportional odds model, known as graded response model in the IRT literature (7,19), where the probability (*P*) of rating a score of at least *k* (*k* = 0 to 4) was described as in Equation 1, and the probability of rating exactly score *k* derived as in Equation 2.

1 $$ P\left({Y}_{ij}\ge k\right)=\frac{1}{1+{e}^{-{a}_j\left({W}_{i,l}-{b}_{j,k}\right)}} $$

2 $$ P\left({Y}_{ij}=k\right)=P\left({Y}_{ij}\ge k\right)-P\left({Y}_{ij}\ge k+1\right) $$

where *a*_*j*_ and *b*_*j*, *k*_ are, respectively, the discrimination and difficulty parameters specific to item *j*, treated as fixed effects in the model. *b*_*j*, *m*_ are constrained to be non-decreasing for increasing scores of the same item (i.e. *b*_*j*, *k* + 1_ ≥ *b*_*j*, *k*_). *W*_*i*, *l*_ is modeled as a random variable on an arbitrary scale (from –∞ to +∞), where higher values denote better well-being. To develop the base IRT model, FACT-B data from each patient and visit were treated independently (assuming no intra-patient correlation between visits). This was done to facilitate the estimation of item-specific parameters, as described below, without having to assume any specific shape of well-being time-course. *W*_*i*, *l*_ was assumed to follow a normal distribution with mean of 0 and variance of 1 at baseline in the T-DM1 arm, and estimated mean and variance at later observations. To account for the possibility that the different aspects of the well-being vary in relation to each other, correlations between *W*_*i*, *l*_ of the different subscales were estimated. Potential deviation from the normality assumption were to be investigated if indicated by the distribution of empirical Bayes estimates (EBEs). Item characteristic curves (ICCs) were generated to illustrate how a patient with a given level of well-being is likely to give a particular answer to each FACT-B item (for each item the probability of each score is plotted against well-being *W*_*l*_). The shape of ICCs is influenced by the item-specific parameters *a*_*j*_ and *b*_*j*, *k*_.

When examining the FACT-B questionnaire, it appeared that BCS items only share their specificity to breast cancer, but each item could be better placed in one of the other subscales (physical, social/family, emotional or functional). In order to explore this hypothesis, an attempt to reassign BCS items to other subscales was made: 1) all FACT-B data were fitted by an IRT model with five *W*_*l*_ variables corresponding to the original FACT-B subscales, 2) the EBEs of *W*_*l*_ obtained from Step 1 for the physical, social/family, emotional and functional subscales were retrieved and incorporated into the dataset, 3) data of each separate BCS item were fitted using Equations 1 and 2, and sequentially using the EBEs of *W*_*l*_ from each of the other subscales, i.e. only estimating *a*_*j*_ and *b*_*j*, *k*_, 4) each BCS item was reassigned to the subscale for which the EBEs of *W*_*l*_ provided the best model fit in step 3 (lowest objective function value, OFV) and all items were modelled simultaneously, estimating all parameters (*W*_*l*_, *a*_*j*_ and *b*_*j*, *k*_).

#### Longitudinal Well-Being Model

A model was developed to describe well-being change over time for each subscale, using the EBEs of *W*_*i*, *l*_ obtained from the base IRT model as dependent variables. Data from different visits were grouped by patient. Linear, power, exponential, Weibull and non-monotonic functions over time were investigated to characterize well-being time-course. Inter-individual variability (IIV) was implemented additively on unconstrained parameters and exponentially on non-negative parameters. In addition, correlations between parameters were explored. In cases where correlations between the same type of parameter on the different subscales were large, a common IIV was estimated for all subscales, together with an additional inter-subscale variability term. Finally, residual unexplained variability in *W*_*i*, *l*_ was described by an additive model. T-DM1 arm data were used to develop the structural model. Capecitabine-plus-lapatinib arm data were then added and the model was refined.

A covariate analysis was thereafter performed to evaluate the effect of potential predictive factors on relevant model parameters. Investigated baseline covariates included patient demographics (age, ethnicity and race), treatment line, Eastern Cooperative Oncology Group (ECOG) performance status, and disease-related factors, including tumor burden category (non-measurable disease *versus* measurable disease with tumor burden < median *versus* measurable disease with tumor burden ≥ median), site of disease involvement (visceral *versus* non-visceral), number of disease sites (≤2 *versus* >2), hormone receptor status (estrogen and/or progesterone receptor positive *versus* both negative), and presence of liver, bone, lung and brain metastases. Additionally, the effect of T-DM1 cycle 1 exposure (AUC_cycle1_ and C_min,cycle1_, treated as continuous or categorized into quartiles) on longitudinal model parameters was evaluated. Covariate analysis was performed using the stepwise covariate model building procedure (SCM), with a significance level of *p* < 0.01 in the forward selection and *p* < 0.001 in the backward elimination. All covariates were first tested on parameters using linear relationships, as described in Equations 3 and 4 for categorical covariates, and Equation 5 for continuous covariates. Piece-wise linear relationships were evaluated for continuous covariates upon inclusion of a linear relationship by the SCM.

3 $$ PRM={\theta}_{PRM}+{\theta}_{COV} $$

4 $$ {\theta}_{COV}=\left\{\begin{array}{c}0\  if\ reference\ category\\ {}{\theta}_{COV,1}\  if\ category\ 1\\ {}{\theta}_{COV,2}\  if\ category\ 2\\ {}\dots \end{array}\right. $$

5 $$ PRM={\theta}_{PRM}+{\theta}_{COV}\cdotp \left( COV- CO{V}_{median}\right) $$

where *θ*_*PRM*_ is the typical value of the unconstrained model parameter PRM, *θ*_*COV*_ is the coefficient for the effect of the covariate (COV) on PRM, and COV_median_ is the median value of COV in the data set. Given the large number of relations to be investigated, and to reduce computation time, sequential SCMs were performed. First, covariate effects on baseline model parameters were investigated, assuming the same covariate effect in both arms and all subscales; this allowed to significantly reduce the number of evaluated relations at each SCM steps compared to testing arm- or subscale-specific effects. Second, statistically significant covariates were tested for additional subscale-specific effects, and all relations were reevaluated in a backward elimination. Third, additional disease-related covariate effects were explored on longitudinal model parameters in an arm-dependent manner. Finally, T-DM1 exposure metrics were tested on longitudinal model parameters in the T-DM1 arm.

#### Longitudinal IRT Model

The base IRT and longitudinal well-being models were combined without re-estimation into a longitudinal IRT model that describes the temporal change in the probability of each score for each item, including potential covariate effects identified in the longitudinal well-being model. Simulation-based diagnostics were computed as detailed in the next section. For each item and covariate value of interest, the typical probabilities of each score were predicted by the model. Moreover, the typical expected score was calculated as the sum of the scores, weighted by their probability.

#### Model Building and Evaluation

Data analysis and simulations were performed using the nonlinear mixed effect software NONMEM version 7.3 (20). The first-order conditional estimation method was used for parameter estimation of the longitudinal well-being model and the second-order conditional estimation with Laplacian approximation for parameter estimation of IRT models. Data pre- and post-processing, model diagnostics and graphical visualization were assisted by R software version 3.1.1, Perl-speaks-NONMEM (PsN) toolkit version 4 and Pirana version 2.9.0 (21).

Model selection was based on graphical diagnostic plots, relative standard errors (RSE) of model parameters from the NONMEM variance-covariance matrix, and OFV used in likelihood ratio tests. For nested models, an OFV difference corresponding to a significance level of *p* < 0.01 was used for model discrimination (6.63 points for models that differ by one parameter). To diagnose the relationship between the latent variable and the response in the IRT model, the estimated cumulative probability curve of each score *versus* the latent variable was compared to the fit of a generalized additive model (GAM) to the data *versus* the EBEs of the latent variable, using a cross-validated cubic spline as a smoothing function (22). The predictive performance of the longitudinal IRT model was assessed through visual predictive checks (VPCs), where for each item the 95% confidence interval (CI) for the average score, obtained from 200 Monte Carlo simulations using the original study design, was compared to the observed average score. Additionally, VPCs of the proportion of each score for each item were examined. Finally, VPCs of the sum of scores for each subscale (calculated as per FACT-B scoring guidelines) were generated, where the 95% CI for the 2.5th, 50th and 97.5th percentiles of the simulated sum of scores were compared to the corresponding observed percentiles.

## Results

### Data

The distribution of observed items’ scores at baseline in the T-DM1 arm showed a variety of patterns, with some items being skewed towards high scores (most items in the physical and social/family subscales), and others being more evenly distributed (most items in emotional and functional subscales) (***Supplementary Fig.*** ***S1***).

### IRT Model

#### Base IRT Model

In the base IRT model, 180 item-specific parameters (*a*_*j*_ and *b*_*j*, *k*_) were estimated using T-DM1 data. The use of four latent well-being variables (*W*_*physical*_, *W*_*social*_, *W*_*emotional*_ and *W*_*functional*_), with each BCS item reassigned to one of the four other subscales as described in the Methods section, resulted in an improved model fit despite the use of fewer model parameters (1138 points OFV reduction compared to the model with five well-being variables that included *W*_*breast cancer*_). Two items were reassigned to the physical well-being subscale (“Short of breath” and “Arms swollen or tender”), five items to the emotional well-being subscale (“Self-conscious about way I dress”, “Bothered by hair loss”, “Worry that family members get the same illness”, “Worry about effect of stress on illness”, “Bothered by change in weight”) and two items to the functional well-being subscale (“Feel sexually attractive”, “Feel like a woman”). OFVs obtained for each item and alternative subscale are provided in ***Supplementary Table*** ***S2***. Visual inspection of the distribution of the EBEs of *W*_*l*_ for all four subscales showed no particular deviation from the standard normal distribution; therefore no alternative distributions were considered.

Item-specific parameter estimates are summarized in ***Supplementary Table*** ***S3***. As illustrated by the ICCs (***Supplementary Fig.*** ***S2***), the items differed in their relation to the latent well-being. Some ICCs were steeper (e.g. “Usually do for fun”), suggesting that the information content is larger than for items with flatter ICCs (e.g. “Sleeping well”) in a given well-being range. Moreover, items with flatter ICCs are less sensitive to changes in well-being. Finally, for some items such as “Emotional support from family”, all curves are located towards low well-being values, meaning that those items will enable to differentiate among patients with poor well-being but not among those with better well-being. GAM-based diagnostic plots are provided in ***Supplementary Fig.*** ***S3***. Eta-shrinkage for all four well-being variables ranged 8.8–16%.

Mean score distributions obtained from simulations from the base IRT model developed on T-DM1 data were compared to observed baseline mean scores in the capecitabine-plus-lapatinib arm (***Supplementary Fig.*** ***S4***). Overall, all items’ mean scores were well described, despite a slight underprediction for “Sex life”, “Sexually attractive” and “Bothered by hair loss”.

#### Longitudinal Well-Being Model

On all four subscales, the time-course of well-being (*W*_*l*_*(t)*) was best described by an asymptotic function of time (Equation 6):

6 $$ {W}_{i,l}(t)={W}_{0,i,l}+{W}_{ss,i,l}\cdot \left(1-{e}^{-\frac{\ln (2)}{T_{1/2,i,l}}\cdot t}\right) $$

Where for each individual *i* and subscale *l*, *W*_0, *i*, *l*_ is the baseline latent well-being, *W*_*ss*, *i*, *l*_ the difference in latent well-being from baseline at steady-state and ${T}_{1/2,i,l}$ the progression half-life, i.e. the time to reach *W*_0, *i*, *l*_ plus half of *W*_*ss*, *i*, *l*_. Well-being could improve (*W*_*ss*, *i*, *l*_ > 0), remain stable (*W*_*ss*, *i*, *l*_ = 0) or worsen (*W*_*ss*, *i*, *l*_ < 0) over time. Of note, Weibull models could fit the data of all subscales statistically significantly better than exponential models (dOFV = 29.9 for four additional parameters) but were numerically unstable and therefore not retained. Fixing *W*_0, *physical*_, *W*_0, *emotional*_ and *W*_0, *functional*_ typical values to zero improved numerical stability without worsening model fit. All fixed and random effects were shared between T-DM1 and capecitabine-plus-lapatinib arms, except *W*_*ss*, *physical*_ and *W*_*ss*, *social*/*family*_ typical values which had arm-specific estimates. Additionally, correlations between *W*_*ss*, *i*, *l*_ were estimated to be large (62–96%); hence, a common IIV term was estimated for all subscales (*ω* = 0.59), together with an inter-subscale variability term (*ω* = 0.16). The latter allowed patients to progress differently on each subscale. The largest difference between study arms was obtained for *W*_*ss*, *physical*_, which was not significantly different from zero, and therefore fixed to zero, in the T-DM1 arm (i.e. well-being typically stayed stable), but was estimated to −0.251 (95% CI: -0.303;-0.199) in the capecitabine-plus-lapatinib arm. The latter value indicates that physical well-being typically worsens from baseline by 0.251 standard deviation. Similarly, *W*_*ss*, *social*/*family*_ worsened in both arms, with a more pronounced progression in T-DM1 arm (−0.244, 95% CI: -0.315; −0.173) compared to capecitabine-plus-lapatinib arm (−0.137, 95% CI: -0.223; −0.0514). In both arms, emotional well-being typically improved over time (*W*_*ss, emotional*_ estimated to 0.295, 95% CI: 0.256; 0.334), whereas functional well-being typically stayed stable (*W*_*ss, functional*_ was not significantly different from zero and was therefore fixed to zero). Subscale-specific *T*_1/2, *l*_ estimates were in the range of 30.7–48.9 days for all subscales except for social/family well-being where it was longer (117 days). A large IIV in *T*_1/2, *l*_ was estimated (121% CV), common to all subscales. Correlations between individual *W*_0_ on different subscales ranged between 44 and 81%. Finally, negative correlations between the IIV in *W*_0, *l*_ and the IIV in *W*_*ss*, *l*_ were estimated (−23 to −33%).

In the covariate analysis, statistically significant effects of race (Asian *versus* non-Asian) and ECOG were identified for baseline well-being *W*_0_. Asian patients and patients with ECOG of 1 typically had less favourable well-being at baseline than non-Asian patients and patients with ECOG of 0, respectively. When evaluated on a subscale level, Asian patients typically had worse baseline social/family (dOFV = −73.6, estimated coefficient of −0.441) and functional (dOFV = −22.7, estimated coefficient of −0.181) well-being compared to non-Asian patients. Estimated coefficients can be interpreted in standard deviation terms on the well-being scale, e.g. a typical Asian patient has a social/family well-being value that is 0.441 standard deviation away toward less favorable well-being as compared to a typical non-Asian patient. The ECOG effect was retained on *W*_0, *physical*_ (dOFV = −97.3) and *W*_0, *functional*_ (dOFV = −64.2), with a shared estimated coefficient of −0.268. None of the other covariate effects was statistically significant on *W*_0_ or *W*_*ss*_ upon inclusion of race and ECOG in the model.

Finally, none of the investigated relations between T-DM1 exposure (AUC_cycle 1_ and C_min, cycle 1_ treated as continuous or binned by quartiles) and well-being progression (subscale-specific *W*_*ss*_) was statistically significant (all *p* > 0.1).

Table I provides the parameter estimates and their uncertainty. All parameters were estimated with reasonable uncertainty (RSE ≤32%).

**Table I:** Parameter Estimates and Their Uncertainty (Relative Standard Error, RSE %) from the Final Longitudinal Item-Response Theory Model

Parameter	T-DM1 arm	Capecitabine-plus-lapatinib arm
W_0, physical_ (unitless)	0 fixed^a^	0 fixed^a^
W_0, social/family_ (unitless)	−0.0901 (23)^a^	−0.0901 (23)^a^
W_0, emotional_ (unitless)	0 fixed^a^	0 fixed^a^
W_0, functional_ (unitless)	0 fixed^a^	0 fixed^a^
W_ss, physical_ (unitless)	0 fixed	−0.251 (11)
W_ss, social/family_ (unitless)	−0.244 (15)	−0.137 (32)
W_ss, emotional_ (unitless)	0.295 (6.8)^a^	0.295 (6.8)^a^
W_ss, functional_ (unitless)	0 fixed	0 fixed
T_1/2,physical_ (days)	30.7 (9.0)^a^	30.7 (9.0)^a^
T_1/2,social/family_ (days)	117 (18)^a^	117 (18)^a^
T_1/2,emotional_ (days)	35.1 (12)^a^	35.1 (12)^a^
T_1/2,functional_ (days)	48.9 (7.4)^a^	48.9 (7.4)^a^
${\omega}_{W_{0, physical}}$ (unitless)	0.76 (2.5)^a^	0.76 (2.5)^a^
${\omega}_{W_{0, social/ family}}$ (unitless)	0.69 (2.5)^a^	0.69 (2.5)^a^
${\omega}_{W_{0, emotional}}$ (unitless)	0.81 (2.6)^a^	0.81 (2.6)^a^
${\omega}_{W_{0, functional}}$ (unitless)	0.79 (2.5)^a^	0.79 (2.5)^a^
${\omega}_{W_{ss, IIV}}$ (unitless)	0.59 (4.9)^a^	0.59 (4.9)^a^
${\omega}_{W_{ss, ISV}}$ (unitless)	0.16 (14)^a^	0.16 (14)^a^
${\upomega}_{{\mathrm{T}}_{1/2,\mathrm{IIV}}}$ (% CV)	121 (4.2)^a^	121 (4.2)^a^
$\uprho \left({\upeta}_{{\mathrm{W}}_{0,\mathrm{physical}}};{\upeta}_{{\mathrm{W}}_{0,\mathrm{social}}}\right)$ (%)	44 (4.7)^a^	44 (4.7)^a^
$\uprho \left({\upeta}_{{\mathrm{W}}_{0,\mathrm{physical}}};{\upeta}_{{\mathrm{W}}_{0,\mathrm{emotional}}}\right)$ (%)	79 (2.9)^a^	79 (2.9)^a^
$\uprho \left({\upeta}_{{\mathrm{W}}_{0,\mathrm{physical}}};{\upeta}_{{\mathrm{W}}_{0,\mathrm{functional}}}\right)$ (%)	81 (2.8)^a^	81 (2.8)^a^
$\uprho \left({\upeta}_{{\mathrm{W}}_{0,\mathrm{social}/\mathrm{family}}};{\upeta}_{{\mathrm{W}}_{0,\mathrm{emotional}}}\right)$ (%)	51 (4.2)^a^	51 (4.2)^a^
$\uprho \left({\upeta}_{{\mathrm{W}}_{0,\mathrm{social}/\mathrm{family}}};{\upeta}_{{\mathrm{W}}_{0,\mathrm{functional}}}\right)$ (%)	77 (3.0)^a^	77 (3.0)^a^
$\uprho \left({\upeta}_{{\mathrm{W}}_{0,\mathrm{emotional}}};{\upeta}_{{\mathrm{W}}_{0,\mathrm{functional}}}\right)$ (%)	69 (3.2)^a^	69 (3.2)^a^
$\uprho \left({\upeta}_{{\mathrm{W}}_{0,\mathrm{physical}}};{\upeta}_{{\mathrm{W}}_{ss,,\mathrm{IIV}}}\right)$ (%)	−33 (8.1)^a^	−33 (8.1)^a^
$\uprho \left({\upeta}_{{\mathrm{W}}_{0,\mathrm{social}/\mathrm{family}}};{\upeta}_{{\mathrm{W}}_{ss,,\mathrm{IIV}}}\right)$ (%)	−23 (11)^a^	−23 (11)^a^
$\uprho \left({\upeta}_{{\mathrm{W}}_{0,\mathrm{emotional}}};{\upeta}_{{\mathrm{W}}_{ss,,\mathrm{IIV}}}\right)$ (%)	−29 (8.6)^a^	−29 (8.6)^a^
$\uprho \left({\upeta}_{{\mathrm{W}}_{0,\mathrm{functional}}};{\upeta}_{{\mathrm{W}}_{ss,,\mathrm{IIV}}}\right)$ (%)	−30 (8.4)^a^	−30 (8.4)^a^
Asian on W_0, social/family_	−0.441 (12)^a^	−0.441 (12)^a^
Asian on W_0, functional_	−0.181 (21)^a^	−0.181 (21)^a^
ECOG 1 on W_0, physical_ and W_0, functional_	−0.268 (11)^a^	−0.268 (11)^a^

W_0, subscale_, subscale-specific baseline latent well-being; W_*ss*, subscale_, difference in subscale-specific latent well-being from baseline at steady-state (progression asymptote), T_1/2_, progression half-life, i.e. the time to reach baseline well-being plus half of the steady-state well-being; ω, standard deviation of the random effects; ρ, correlation; IIV, inter-individual variability; ISV, inter-subscale variability; ECOG, Eastern Cooperative Oncology Group functional status

^a^ Common parameter estimated for both study arms

#### Longitudinal IRT Model

**Fig. 2:**
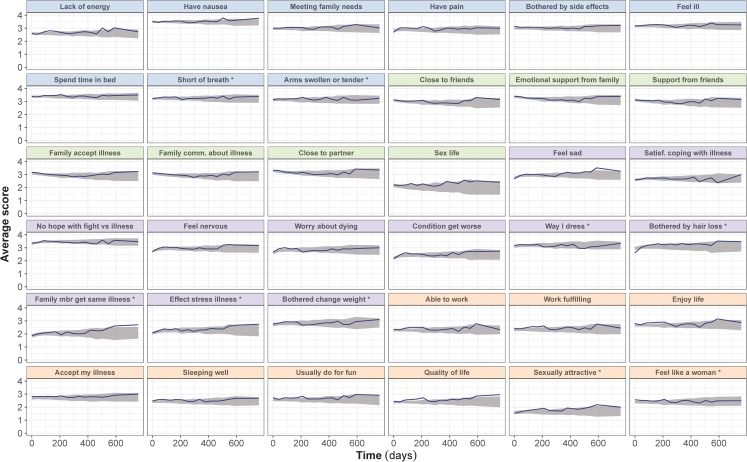
Visual predictive checks of the average score from the final longitudinal item response theory model, stratified by FACT-B items, in the ado-trastuzumab emtansine (T-DM1) arm. The observed average score (solid line) is compared to the 95% confidence interval (shaded area) of the average score over time, obtained from 200 simulations. Scores for reverse items have been reverse-scaled (i.e. higher score indicates better outcome). Panels’ color corresponds to the item subscale: blue for physical, green for social/family, purple for emotional and orange for functional. * Items originally belonging to the breast-cancer subscale.

**Fig. 3:**
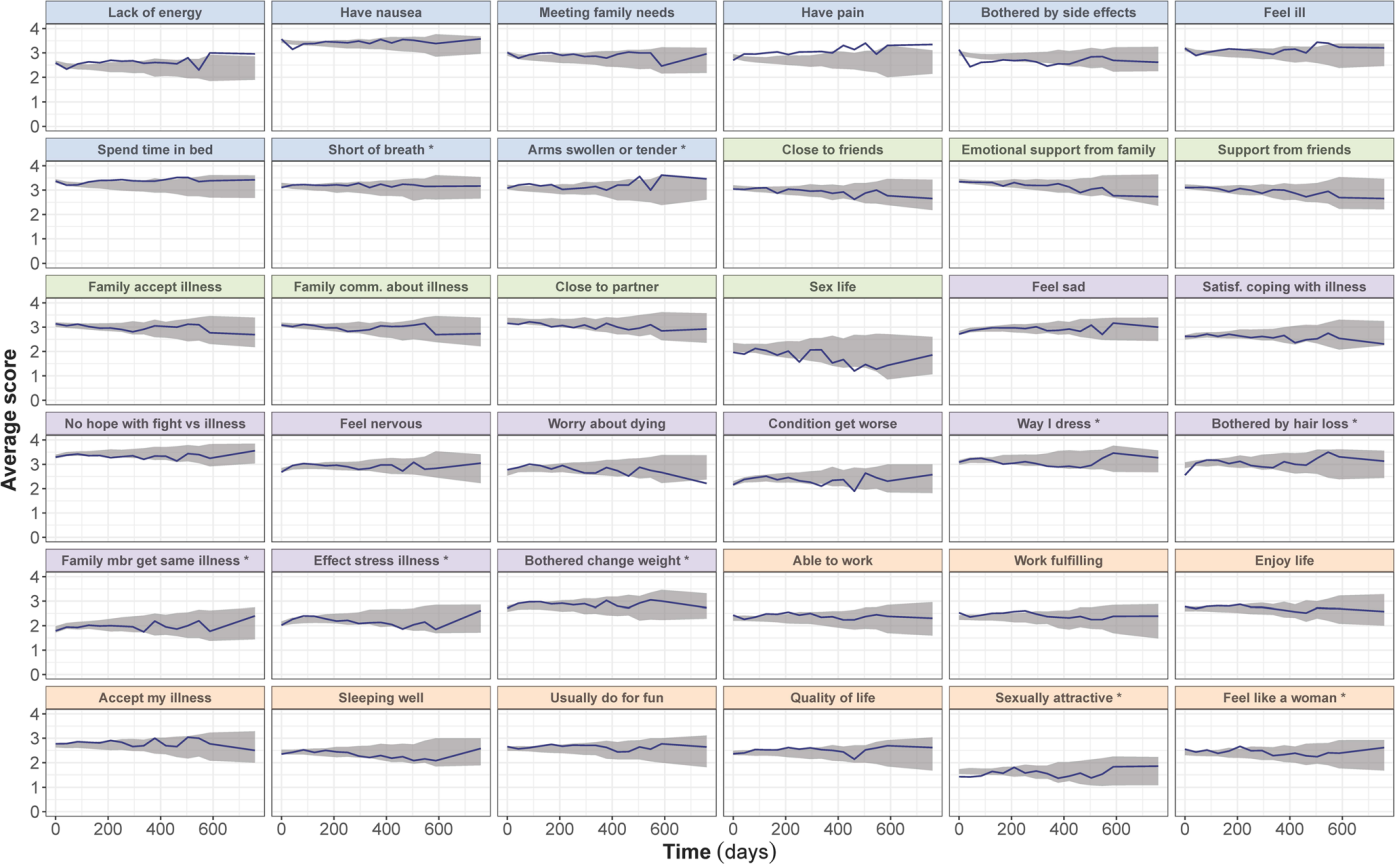
Visual predictive checks of the average score from the final longitudinal item-response theory model, stratified by FACT-B items, in the capecitabine-plus-lapatinib arm. The observed average score (solid line) is compared to the 95% confidence interval (shaded area) of the average score over time, obtained from 200 simulations. Scores for reverse items have been reverse-scaled (i.e. higher score indicates better outcome). Panels’ color corresponds to the item subscale: blue for physical, green for social/family, purple for emotional and orange for functional. * Items originally belonging to the breast-cancer subscale.

**Fig. 4:**
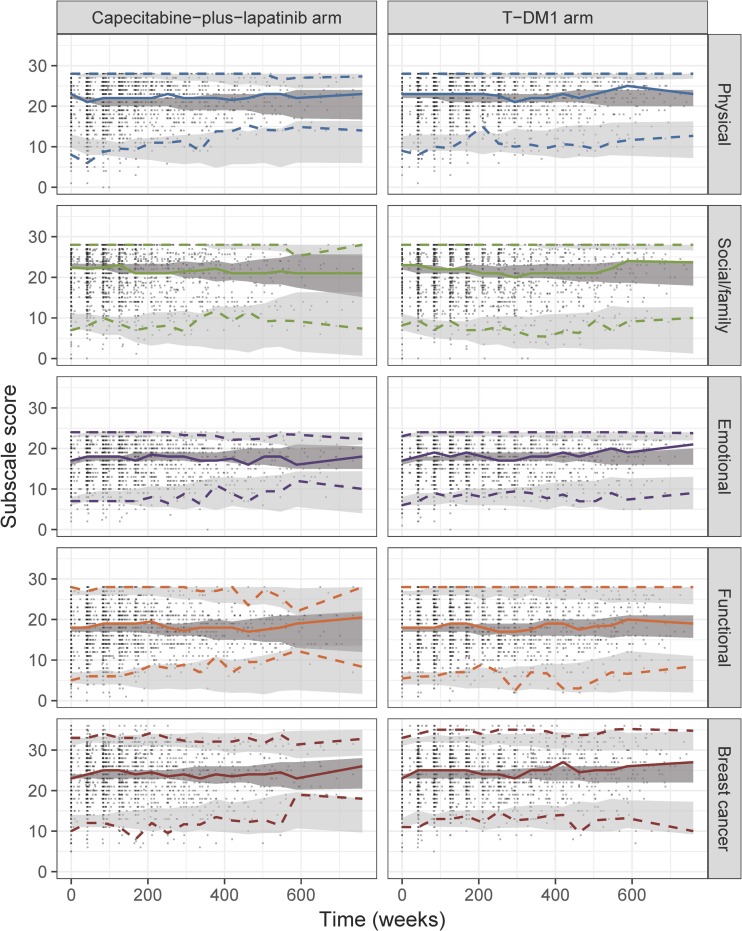
Visual predictive checks of the total subscale score time-course from the final longitudinal item-response theory model, stratified by study arm and subscale (as defined in FACT-B). The median (solid line), 2.5th and 97.5th percentiles (dashed lines) of the observed data are compared to the 95% confidence intervals (shaded areas) for the corresponding percentiles of the simulated data (based on 200 simulations). T-DM1: ado-trastuzumab emtansine.

**Fig. 5:**
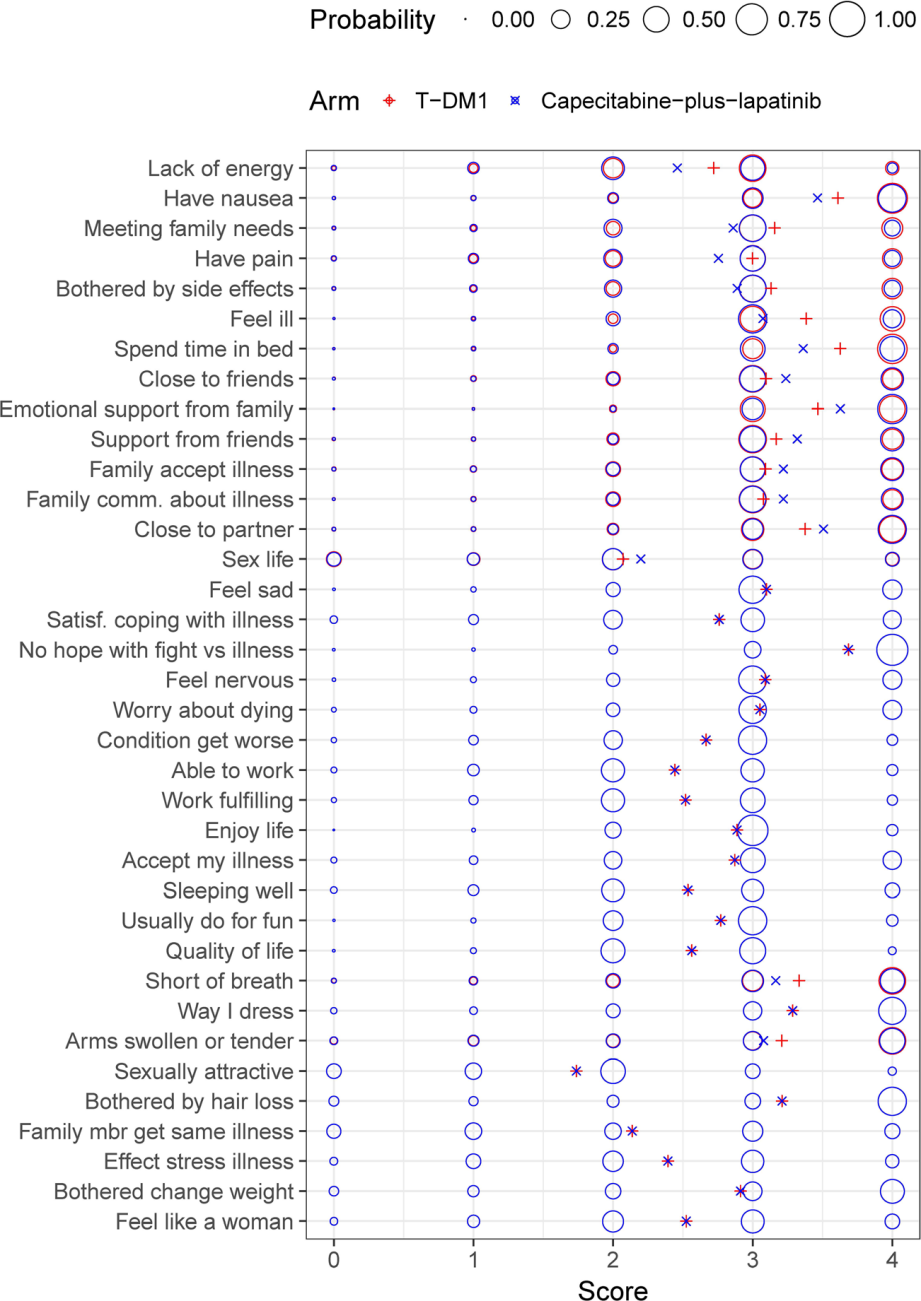
Schematic representation of the item score probabilities and expected score at steady-state for a typical non-Asian patient with baseline ECOG of 0, as predicted by the final longitudinal item-response theory model and differentiated by treatment arm. Circle surface areas are proportional to the score probability. Cross symbols represent expected scores, calculated as $\sum \limits_{k=0}^4P\left(Y=k\right)\cdotp k$. Scores for reverse items have been reverse-scaled (i.e. higher score indicates better outcome). For items where no difference between treatment arms was identified, circles overlap and only one circle can be seen. T-DM1: ado-trastuzumab emtansine.

The longitudinal well-being model was integrated into the IRT framework. As shown by the item-level VPCs, the final longitudinal IRT model was able to satisfactorily predict the average item scores in both the T-DM1 arm (Fig. 2) and in the capecitabine-plus-lapatinib arm (Fig. 3), despite underprediction of “Having pain” item in the capecitabine-plus-lapatinib arm. In addition, VPCs of the sum of scores in each original FACT-B subscale (Fig. 4) indicate that the model adequately predicts the median trend over time and the variability in both treatment arms, although at some early time points the 2.5th percentile tends to be overpredicted in the physical and breast cancer subscale. Figure 5 illustrates the difference in steady-state probabilities and expected scores between the two arms. These differences were largest for items in the physical well-being subscale. In addition, to illustrate the effect of race and ECOG on the items’ scores at baseline, typical expected scores are provided in ***Supplementary Figs. S5 and S6***. The NONMEM code and an example data set is provided as supplementary material.

## Discussion

The presented analysis is the first application of an IRT pharmacometric approach to describe PRO data collected in a cancer clinical study. To ease model building and facilitate parameter estimation, a new stepwise approach was adopted, where the latent well-being estimates obtained from each patient’s visit in a first step were used as dependent variables to build the longitudinal model. The developed IRT model adequately described the time-course of FACT-B in T-DM1 and capecitabine-plus-lapatinib arms from a phase III clinical trial. It could characterize FACT-B data both at the item level and the subscale level. To our knowledge, this is also the first attempt to relate drug exposure to PRO.

For PROs, IRT has previously been described as a useful tool to improve the development and refinement of questionnaires. Mixed models from item response theory have also been shown to be particularly suitable for the analysis of longitudinal HRQoL questionnaire data from cancer clinical trials (23). When integrated into a pharmacometric framework, IRT has demonstrated superior power in detecting drug effect when compared to the analysis of composite scores (e.g. sum of scores) for several neurological diseases (9,13). Using the IRT pharmacometric approach to assess T-DM1, no statistically significant relationship was identified in the T-DM1 arm between FACT-B response and any of the investigated exposure metrics (model-derived cycle 1 C_min_ or AUC, treated as continuous or categorized into quartiles) at the investigated dosing schedule of 3.6 mg/kg q3w. These results are consistent with a previous analysis of the EMILIA study data by Li *et al.*, where no meaningful exposure-response relationship was observed for any safety endpoints (any grade ≥ 3 treatment-related adverse event, grade ≥ 3 thrombocytopenia and grade ≥ 3 hepatotoxicity) (24). In contrast, an exposure-response trend was identified for platelet count and liver enzyme time-courses when modelled on a continuous scale in T-DM1-treated metastatic breast cancer patients (25), but both thrombocytopenia and hepatotoxicity are generally asymptomatic. In addition, in the analysis of efficacy by Li *et al.*, a higher model-predicted cycle 1 C_min_ was associated with longer median overall survival and progression-free survival, findings which were in line with findings by the US FDA during the biologic license application review (17). However, these relationships were not consistent across exposure metrics and were thought to be confounded by baseline disease risk factors. These previous findings suggested that dose adjustment in patients with low exposure at the approved starting dose is not warranted. Our results indicate that lower T-DM1 exposure in the range of evaluated doses would not come at the expense of patient’s well-being, as measured by FACT-B.

The suggested IRT pharmacometric approach has multiple advantages over statistical analysis based on sum of scores. IRT focuses on the individual items, whereas a summary score-based approach typically disregards data at the item-level, resulting in a loss of information. Summing item scores is legitimate when the items contain a similar proportion of information concerning the underlying construct being measured (26). As suggested by the item characteristic curves, FACT-B items contained varying information with regard to the latent well-being and IRT analysis is therefore considered advantageous. Importantly, IRT allows predictors, such as patient characteristics and drug exposure, to affect all items within a subscale through a unique effect on the latent variable. The extent to which each item is affected depends on its relationship to the latent variable (visualized on item characteristic curves) in the latent variable range of interest. IRT models are therefore halfway between methods based on sum of scores and those considering separate predictor effects on each item, which are not practically applicable to scales with large number of items (8).

In addition, by pooling data from multiple subjects in a non-linear mixed effect framework, the present approach has advantages over traditional statistical methods when analyzing data for fixed time points (scheduled visits), which are particularly sensitive to missed or delayed assessments (27). In our analysis, missing data (i.e. missing visits) were assumed to occur at random or completely at random and therefore ignorable. In cases where informative dropout is suspected and missing data are not at random, the IRT model may be combined with logistic regression models that would describe dropout patterns (11,28). Additionally, item responses that are missing completely at random can be ignored with no need for imputation; more advanced techniques to model non-ignorable missingness are available in IRT literature (29). Finally, in cases where exposure-response relationships are identified, IRT pharmacometric models allow for simulations to be performed to guide selection of dosing regimens that would maximize PROs. While IRT pharmacometric models are promising tools to address limitations of commonly used statistical methods, mathematical complexity may be perceived as limiting factors to their application. However, it is expected that community efforts to share technical knowledge (e.g. model codes) will ease their application in the future.

The simultaneous analysis of all items in FACT-B in present work also increased our understanding of the structure of the questionnaire. The FACT-B questionnaire consists of the multidimensional FACT-General questionnaire and the BCS, which is meant to complement the general scale by addressing concerns relevant and specific to breast cancer patients. BCS was reported in the literature to have lower internal consistency (as measured by the Cronbach’s alpha coefficient ranging 0.26 to 0.67 in factor analyses) than the other FACT-B subscales, meaning that the BCS is somewhat heterogeneous (30–34). Yoo *et al.* identified three factors in the BCS, namely psychological distress (five items), feminine satisfaction (two items) and physical complaints (two items), thus confirming the multidimensional nature of BCS (31). In our analysis, we reassigned each BCS item to one of the other subscales in a systematic manner, based on objective goodness of fit criteria (OFV). Reassignment was important to satisfy the IRT prerequisite that all items in a subscale should measure a single common underlying construct. Two items were reassigned to the physical well-being, five items to the emotional well-being and two items to the functional well-being subscale, while no item was reassigned to the social/family well-being subscale. These findings are in agreement with results from statistical analyses of the English and the Korean version of FACT-B, both showing statistically significant correlations between BCS score and the scores on physical, emotional and functional subscale scores (0.39–0.61), but lower correlation between BCS and social/family subscale (0.09–0.37) (30,31,33). In addition, while correlations between items within a subscale are inherently accounted for through the latent well-being variable, additional correlations between well-being in the different subscale were estimated. The estimated values (44–81%) are in agreement with previous reports, with the lowest correlation being between physical and social/family subscales (30).

In the covariate analysis, Asian patients were found to exhibit less favorable baseline social/family and functional well-being than non-Asian patients. One could argue that differences may exist in the way patients with different cultural background answer the questions (also known as differential item functioning). However, good psychometric properties have generally been proven for FACT-B English version and several of its translations, including Chinese, Korean, Malayalam and Thai versions. All versions underwent rigorous linguistic validation to ensure content and conceptual equivalence (30–32,34–36). Hence, racial variations in FACT-B are not likely attributable to different item properties across populations, but rather to factors that would affect the underlying well-being, for instance differences in socio-economic status or in seeking social support. Additionally, the current analysis showed that lower ECOG performance status (0 *versus* 1) was associated with better baseline physical and functional well-being, while no relationship was found between ECOG and emotional or social/family well-being. Likewise, Brady *et al.* showed that correlations between ECOG and FACT-B scores were the strongest in the physical and functional domains. Interestingly, the current analysis showed no correlation between well-being and baseline disease-related factors, including tumor burden, number of disease sites, sites of disease involvement and metastases location. In accordance with these results, two separate studies showed that quality of life was not affected by stage of cancer and extent of disease (37,38).

In the longitudinal IRT model, the asymptotic exponential progression function allowed patients’ well-being to improve, worsen or stay stable during the course of therapy. Negative correlations were estimated between the IIV term for baseline and steady-state well-being, indicating that patients with poorer well-being at baseline tend to progress toward better well-being, and vice-versa. A covariate search was undertaken to explain the substantial variability estimated on steady-state well-being, both between patients and between subscales. None of the baseline disease-related factors was found statistically significant (*p* > 0.01). There was however a signal in the data that a larger number of disease sites at baseline was associated with a more favorable well-being progression (*p* = 0.012). In addition, we investigated differences in steady-state well-being between treatment arms. Functional well-being typically stayed stable, whereas emotional well-being improved to the same extent in both arms. Differences were identified in steady-state physical well-being, with more favorable progression in the T-DM1 arm, which translated into worse expected scores in the capecitabine-plus lapatinib arm, as illustrated in Fig. 5 for a typical patient. In an exploratory analysis of EMILIA FACT-B data, Welslau *et al.* found that T-DM1-treated patients were less bothered by side effects of treatment than capecitabine-plus-lapatinib-treated patients through week 24, while differences in other physical items were not statistically or clinically significant (39). Finally, we also found that social/family well-being typically worsened less in patients in the capecitabine-plus-lapatinib arm compared to those in the T-DM1 arm. Reasons for this finding are unclear. Noticeably, social/family well-being progression (estimated half-life of approximately 5.6 cycles) was typically much slower than on other subscales; social/family well-being steady-state estimates should be interpreted with caution as steady-state might occur beyond the observed time-frame.

Potential limitations related to EMILIA study design should be considered when interpreting the results. The differences identified between T-DM1 and capecitabine-plus-lapatinib arms should be interpreted with caution as PRO reporting might have been affected by the open-label nature of the study (40). However, due to practical and ethical reasons open-label designs are very common in oncology, and to date no evidence exists that such bias exists (41). Moreover, differences in the dosing schedule and pharmacokinetic properties between T-DM1 and active control treatment may result in temporal differences in occurrence of adverse effects and symptoms. PRO-time profiles may therefore be sensitive to the timing of reporting (here on day 1 of every odd cycle, before drug administration). In addition, some limitations of the present exposure-response analysis should be noted. First, the range of exposure (AUC_cycle 1_ and C_min,cycle 1_) was limited due to the single dosing regimen of T-DM1 (3.6 mg/kg q3w), which may limit the ability of the model to detect an exposure-response relationship. Cycle 1 maximum concentration was not included in this analysis since it was associated with less variability than C_min,cycle 1_ (18) and was occurring 3 weeks before FACT-B assessment. Second, even though cycle 1 exposure may be considered to be at steady-state given T-DM1 elimination half-life of approximately four days (18), it does not account for potential dose adjustments at later cycles, and underestimation of longitudinal pharmacokinetic variability may be possible. Finally, limitations of the stepwise approach, where EBEs of well-being from the first step were used to build the longitudinal well-being and IRT models, should be noted. Similar to sequential PK-PD approaches, this approach might suffer from higher bias and imprecision compared to simultaneous modeling (42,43). In our analysis, data were rich and eta-shrinkage in the first step was low for all latent well-being variables (≤16%) which likely limit those risks.

## Conclusion

The developed longitudinal IRT model adequately described FACT-B questionnaire data in metastatic and locally advanced breast cancer patients treated with T-DM1 or capecitabine-plus-lapatinib. Through the use of four correlated latent well-being variables, it acknowledges the multi-dimensional nature of the questionnaire and allows a thorough description of the data, not only at the subscale level but also at the item level. No relationship was identified between T-DM1 exposure and any of the latent variables, but differences between treatment arms were observed in the physical and social/family well-being domains. This modeling framework can complement summary score-based approaches, and may be adapted to analyze data from other PRO instruments commonly used in clinical trials or routine clinical practice. The analysis of PRO data combined with traditional efficacy and safety analyses offer all healthcare stakeholders a patient-centric assessment of the impact of a disease and therapy and evidence regarding the overall patient experience of a new drug.

## ACKNOWLEDGMENTS AND DISCLOSURES

This work was supported by Genentech, Inc./F. Hoffman-La Roche, Basel, Switzerland. M.O. Karlsson, L.E. Friberg and E. Schindler have received research grants from Genentech. L.E. Friberg acted as a paid consultant to Genentech. C. Li, B. Wang, B. Lum, A. Quartino, J.Y. Jin, and S. Girish are salaried employees of Genentech, Inc. in South San Francisco and own stock in Roche Holding Ltd. The authors thank Jonathan Squire for data management and Sebastian Ueckert for his valuable input.

## Electronic Supplementary Material

ESM 1 (PDF 1676 kb)

ESM 2 (PDF 308 kb)

## Abbreviations

AUC_cycle 1_: Cycle 1 area under the curve
BCS: Breast cancer subscale
CI: Confidence interval
C_min,cycle1_: Cycle 1 minimum concentration
dOFV: Difference in objective function value
EBE: Empirical Bayes estimate
ECOG: Eastern Cooperative Oncology Group
FACT-B: Functional assessment of cancer therapy-breast
FACT-G: Functional assessment of cancer therapy-general
HER2: Human epidermal growth factor receptor 2
HRQoL: Health related quality of life
ICC: Item characteristic curve
IIV: Inter-individual variability
IRT: Item response theory
OFV: Objective function value
PRO: Patient-reported outcome
RSE: Relative standard error
SCM: Stepwise covariate model building strategy
T-DM1: Ado-trastuzumab emtansine
VPC: Visual predictive check
W: Well-being
