# Supplementary material for: A Pharmacometric Analysis of Patient-Reported Outcomes in Breast Cancer Patients Through Item Response Theory
Source: Pharm Res. 2018 Apr 19;35(6):122. doi: 10.1007/s11095-018-2403-8 (PMC5908825; doi:10.1007/s11095-018-2403-8)
Supplement: Supplementary file 1 — (PDF 1676 kb) [file 11095_2018_2403_MOESM1_ESM.pdf]

Supplementary material: Tables and figures

Table S1: Questions included in the functional assessment of cancer therapy- breast (FACT-B) questionnaire, abbreviated label and information on reverse items

Subscale	FACT-B item	Abbreviated label	Reverse item?	Item number
**Physical well-being**	I have a lack of energy	Lack of energy	Yes	1
	I have nausea	Have nausea	Yes	2
	Because of my physical condition, I have trouble meeting the needs of my family	Meeting family needs	Yes	3
	I have pain	Have pain	Yes	4
	I am bothered by side effects of treatment	Bothered by side effects	Yes	5
	I feel ill	Feel ill	Yes	6
	I am forced to spend time in bed	Spend time in bed	Yes	7
**Social/family well-being**	I feel close to my friends	Close to friends	No	8
	I get emotional support from my family	Emotional support from family	No	9
	I get support from my friends	Support from friends	No	10
	My family has accepted my illness	Family accept illness	No	11
	I am satisfied with family communication about my illness	Family comm. about illness	No	12
	I feel close to my partner (or the person who is my main support)	Close to partner	No	13
	I am satisfied with my sex life	Sex life	No	14
**Emotional well-being**	I feel sad	Feel sad	Yes	15
	I am satisfied with how I am coping with my illness	Satisf. coping with illness	No	16
	I am losing hope in the fight against my illness	No hope with fight vs illness	Yes	17
	I feel nervous	Feel nervous	Yes	18
	I worry about dying	Worry about dying	Yes	19
	I worry that my condition will get worse	Condition get worse	Yes	20
**Functional well-being**	I am able to work (include work at home)	Able to work	No	21
	My work (include work at home) is fulfilling	Work fulfilling	No	22
	I am able to enjoy life	Enjoy life	No	23
	I have accepted my illness	Accept my illness	No	24
	I am sleeping well	Sleeping well	No	25
	I am enjoying the things I usually do for fun	Usually do for fun	No	26

Subscale	FACT-B item	Abbreviated label	Reverse item?	Item number
	I am content with the quality of my life right now	Quality of life	No	27
**Breast cancer subscale**	I have been short of breath	Short of breath	Yes	28
	I am self-conscious about the way I dress	Way I dress	Yes	29
	One or both of my arms are swollen or tender	Arms swollen or tender	Yes	30
	I feel sexually attractive	Sexually attractive	No	31
	I am bothered by hair loss	Bothered by hair loss	Yes	32
	I worry that other members of my family might someday get the same illness I have	Family mbr get same illness	Yes	33
	I worry about the effect of stress on my illness	Effect stress illness	Yes	34
	I am bothered by a change in weight	Bothered change weight	Yes	35
	I am able to feel like a woman	Feel like a woman	No	36
	I have certain parts of my body where I experience pain	Not included - Not currently scored		-

Table S2: Objective function values (OFV) obtained for each breast cancer subscale items when reassigning them to each of the other subscale.

Item	Subscale	OFV	Difference in OFV from best
**Short of breath**	Physical	5475	0
	Social/family	6001	526
	Emotional	5755	280
	Functional	5858	383
**Self-conscious about way I dress**	Physical	6038	77
	Social/family	6210	249
	Emotional	5961	0
	Functional	6139	178
**Arms swollen or tender**	Physical	6147	0
	Social/family	6373	226
	Emotional	6270	123
	Functional	6336	189
**Feel sexually attractive**	Physical	7325	473
	Social/family	7239	387
	Emotional	7366	514
	Functional	6852	0
**Bothered by hair loss**	Physical	6201	165
	Social/family	6284	248
	Emotional	6036	0
	Functional	6241	205
**Worry that family members get the same illness**	Physical	8209	377
	Social/family	8347	515
	Emotional	7832	0
	Functional	8299	467
**Worry about effect of stress on illness**	Physical	7603	583
	Social/family	7914	894
	Emotional	7020	0
	Functional	7801	781
**Bothered by change in weight**	Physical	7231	64
	Social/family	7381	214
	Emotional	7167	0
	Functional	7326	159
**Feel like a woman**	Physical	7418	626
	Social/family	7104	312
	Emotional	7427	635
	Functional	6792	0

Table S3: Item-specific parameter estimates from the final longitudinal item-response theory model.

Item	a	b_1	δ_2	δ_3	δ_4
Lack of energy	2.07	-2.32	0.973	1.02	1.27
Have nausea	1.47	-4.00	1.02	1.16	1.27
Meeting family needs	2.56	-2.25	0.689	0.876	0.977
Have pain	1.75	-2.71	1.04	0.964	1.23
Bothered by side effects	2.07	-2.70	0.879	0.991	1.21
Feel ill	3.15	-2.46	0.737	0.871	0.914
Spend time in bed	2.83	-2.63	0.765	0.709	0.913
Close to friends	3.04	-2.18	0.630	0.719	0.831
Emotional support from family	4.68	-2.10	0.478	0.593	0.735
Support from friends	3.54	-1.85	0.358	0.623	0.807
Family accept illness	2.45	-2.25	0.566	0.793	0.891
Family comm. about illness	3.13	-2.18	0.596	0.780	0.821
Close to partner	2.88	-2.01	0.350	0.640	0.698
Sex life	1.49	-1.42	0.465	0.979	1.04
Feel sad	2.19	-2.47	0.983	1.09	1.21
Satisf. coping with illness	0.830	-3.73	1.38	1.95	2.19
No hope with fight vs illness	2.06	-2.68	0.611	1.04	0.876
Feel nervous	2.04	-2.23	0.794	0.939	1.37
Worry about dying	2.18	-1.69	0.571	0.740	1.21
Condition get worse	2.43	-1.36	0.683	0.731	1.35
Able to work	2.03	-1.96	0.925	1.05	1.17
Work fulfilling	2.62	-1.65	0.648	0.952	1.04
Enjoy life	4.27	-1.94	0.735	0.864	0.893
Accept my illness	1.69	-2.34	0.763	1.04	1.20
Sleeping well	1.43	-2.56	1.06	1.46	1.23
Usually do for fun	3.76	-1.80	0.689	0.885	0.844
Quality of life	3.63	-1.74	0.684	0.996	0.953
Short of breath	1.19	-3.91	1.28	1.21	1.30
Way I dress	0.835	-4.13	1.18	1.62	1.40
Arms swollen or tender	0.765	-4.59	1.64	1.23	1.49
Sexually attractive	1.41	-1.22	0.776	1.44	1.23
Bothered by hair loss	0.840	-2.82	0.796	0.854	1.00
Family mbr get same illness	1.06	-1.33	1.08	0.766	1.40
Effect stress illness	1.51	-1.80	1.21	0.963	1.29
Bothered change weight	0.764	-3.28	1.32	1.31	1.38
Feel like a woman	1.52	-2.20	0.975	1.12	1.25

$b_2 = b_1 + \delta_2; b_3 = b_2 + \delta_3;\ b_4 = b_3 + \delta_4$. See also NONMEM code provided as Supplementary material.

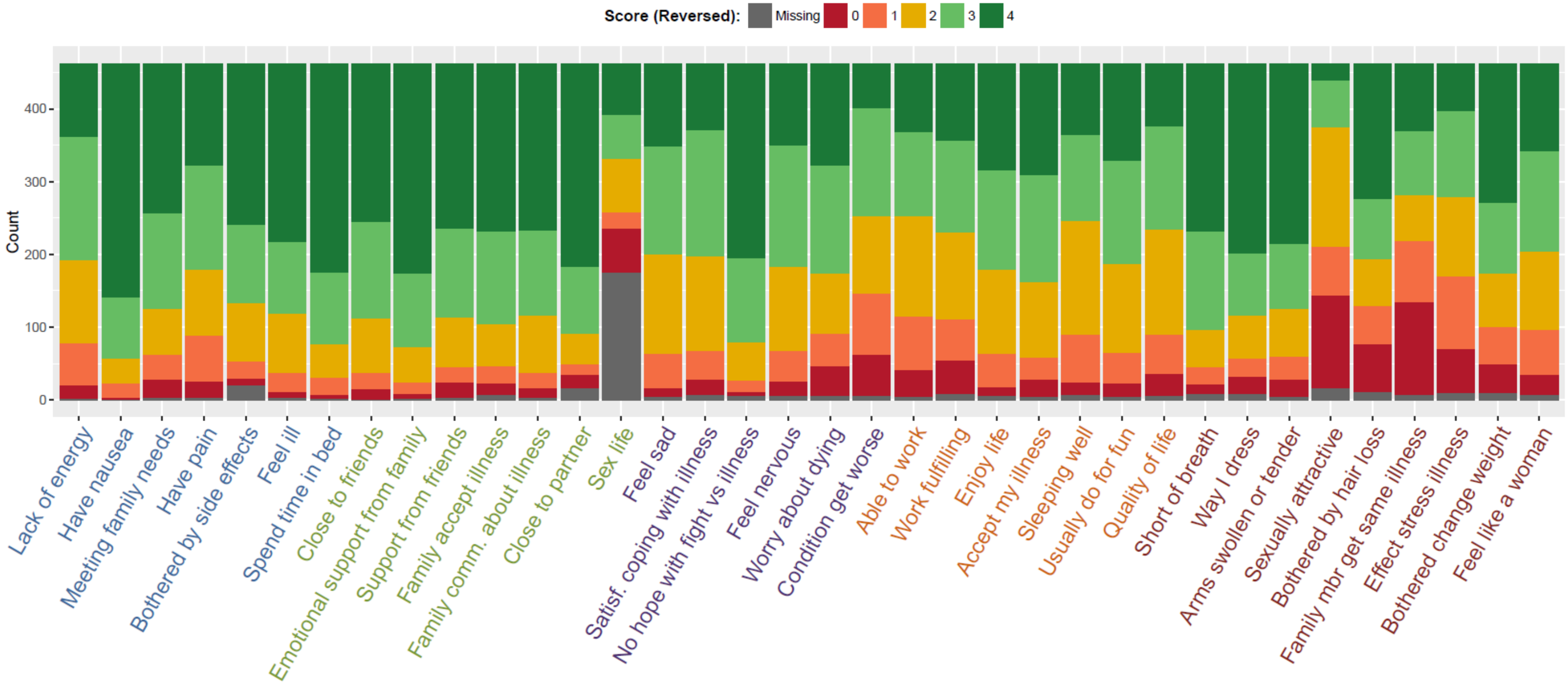

Figure S1: Distributions of observed FACT-B scores at baseline in T-DM1 arm. Label color corresponds to the item's subscale: blue for physical, green for social/family, purple for emotional, orange for functional and red for breast-cancer. Scores for reverse items have been reverse-scaled (i.e. higher score indicate better outcome)

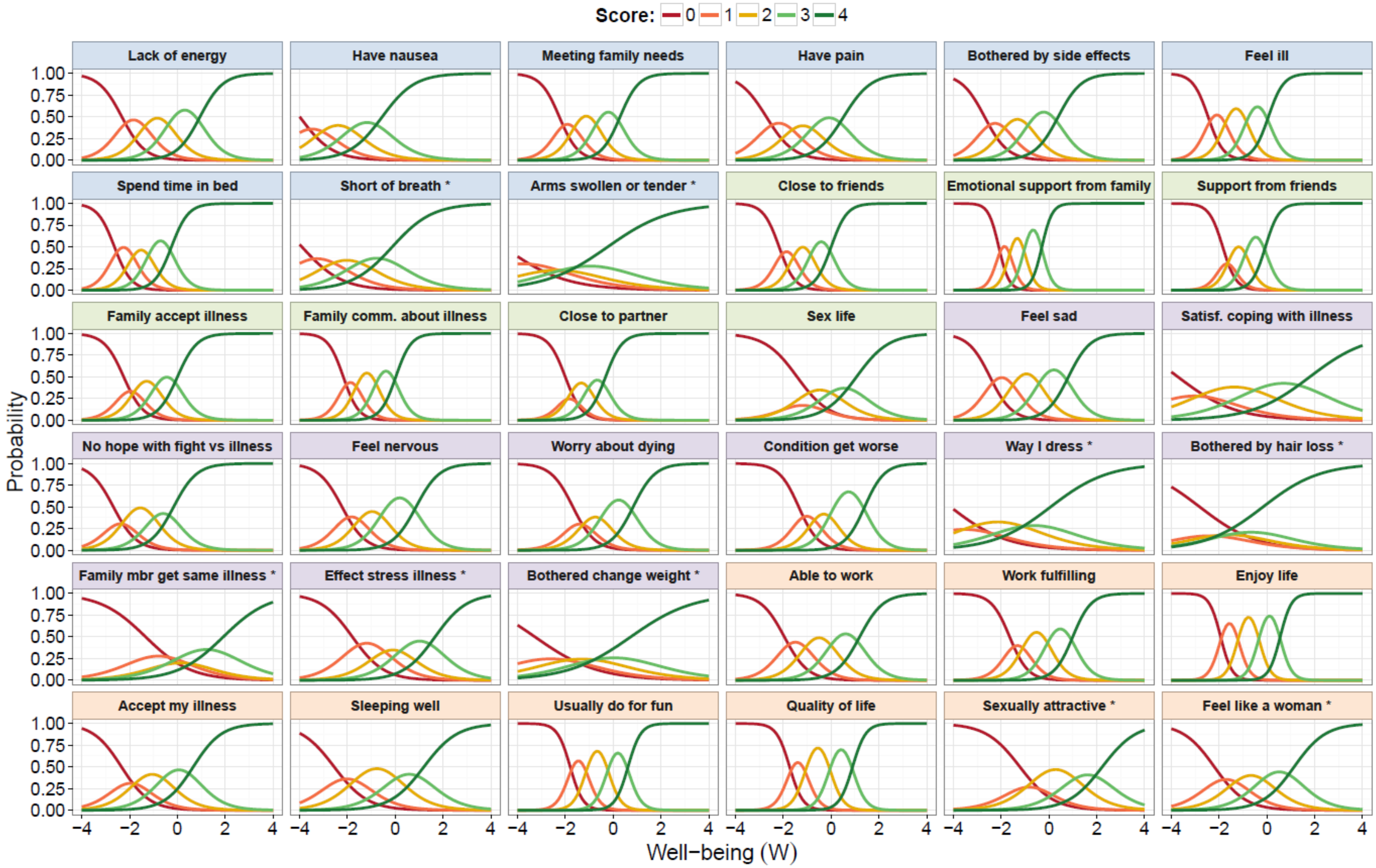

Figure S2: Item characteristic curves obtained from the final longitudinal item-response theory model. For each FACT-B item, the probability of each score is plotted against the latent well-being variable W. Panels' color correspond to the item subscale: blue for physical, green for social/family, purple for emotional and orange for functional. Scores for reverse items have been reverse-scaled (i.e. higher score indicate better outcome). * Items originally belonging to the breast-cancer subscale.

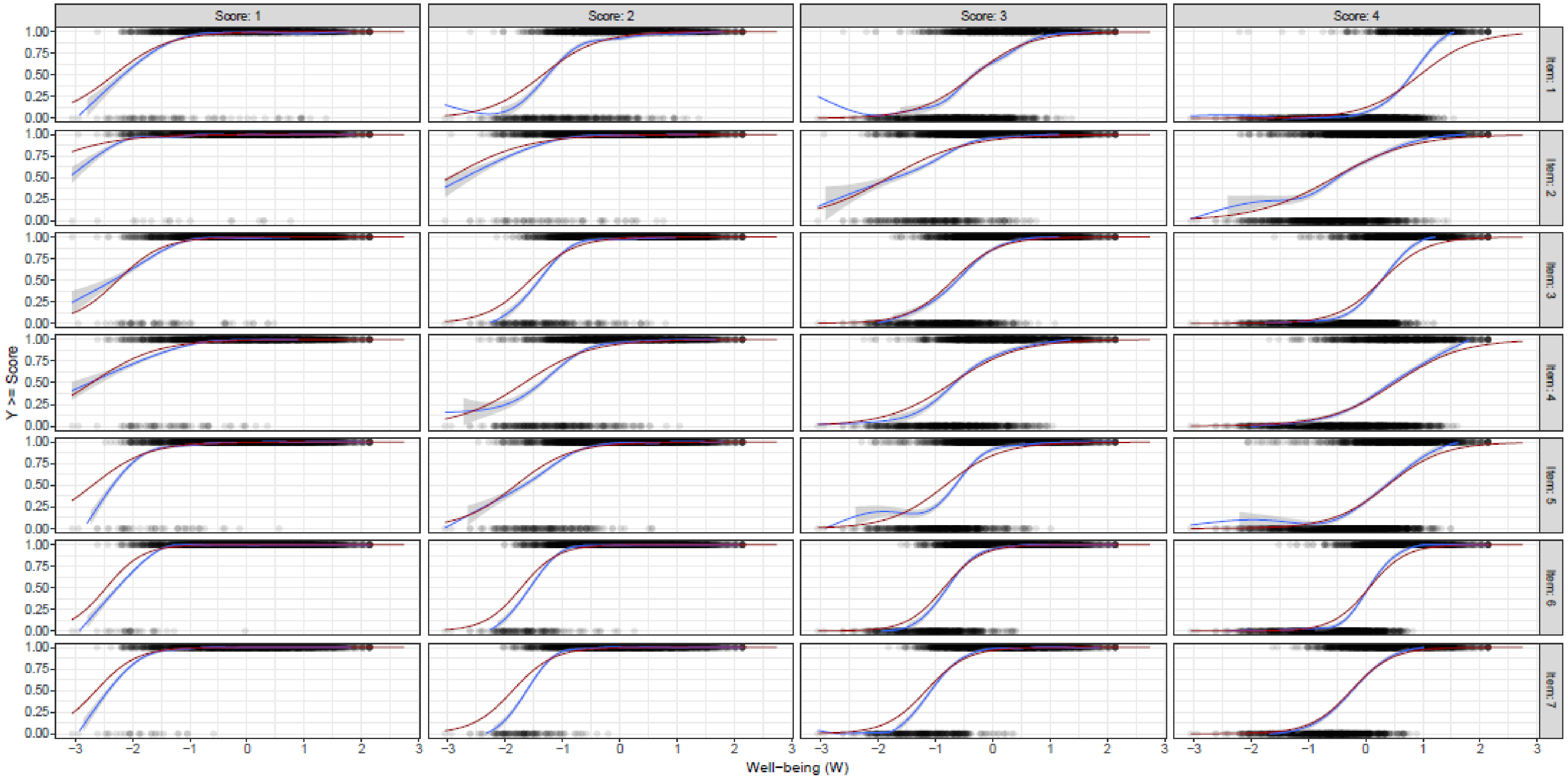

Figure S3: Diagnostic plots comparing the IRT model fit (red line) to the fit of generalized additive model (GAM) with cross-validated cubic spline as a smoothing function (blue line with 95% confidence interval in grey). Item numbers are as described in Table S1.

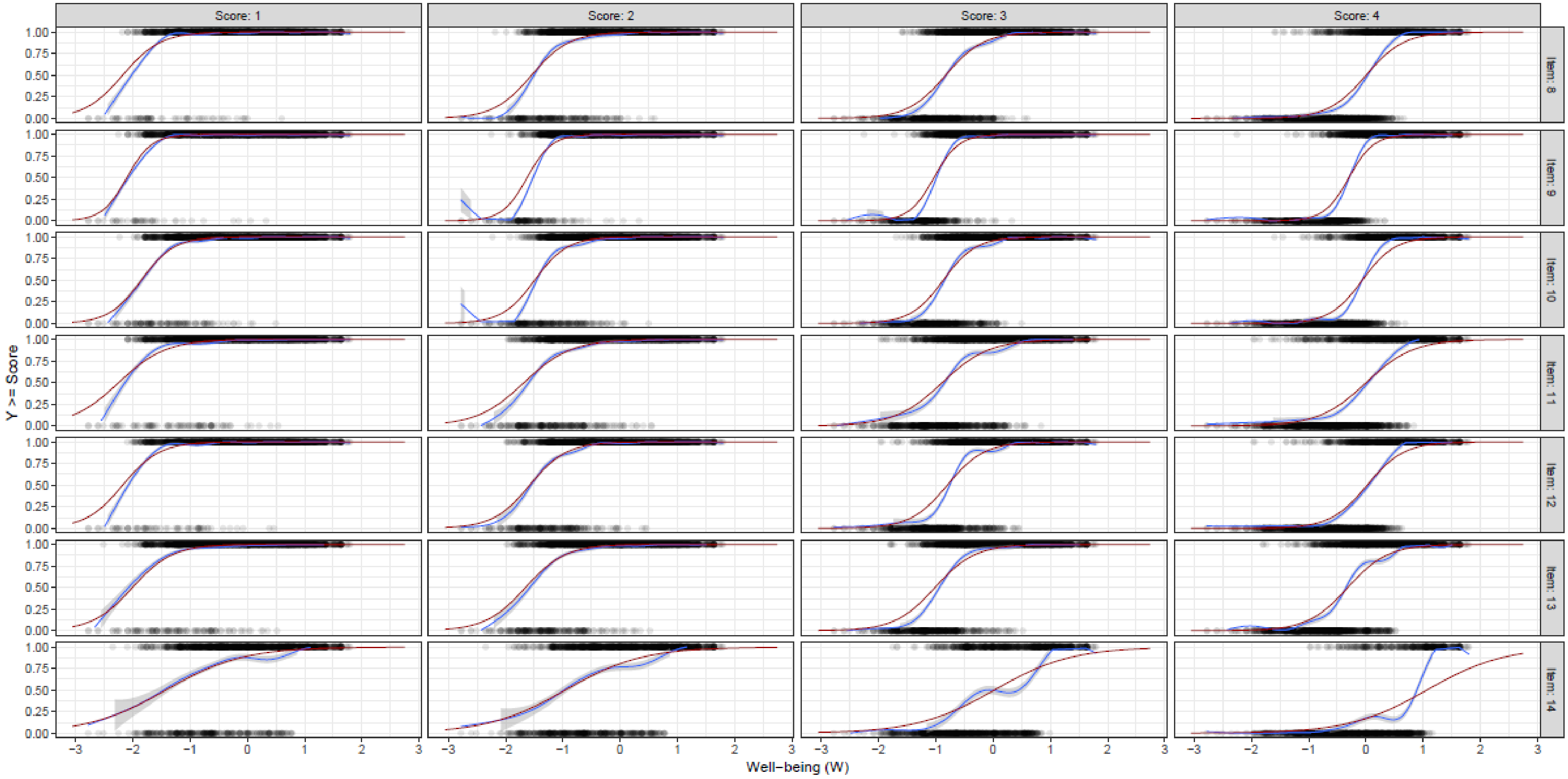

Figure S3 (continued): Diagnostic plots comparing the IRT model fit (red line) to the fit of generalized additive model (GAM) with cross-validated cubic spline as a smoothing function (blue line with 95% confidence interval in grey). Item numbers are as described in Table S1.

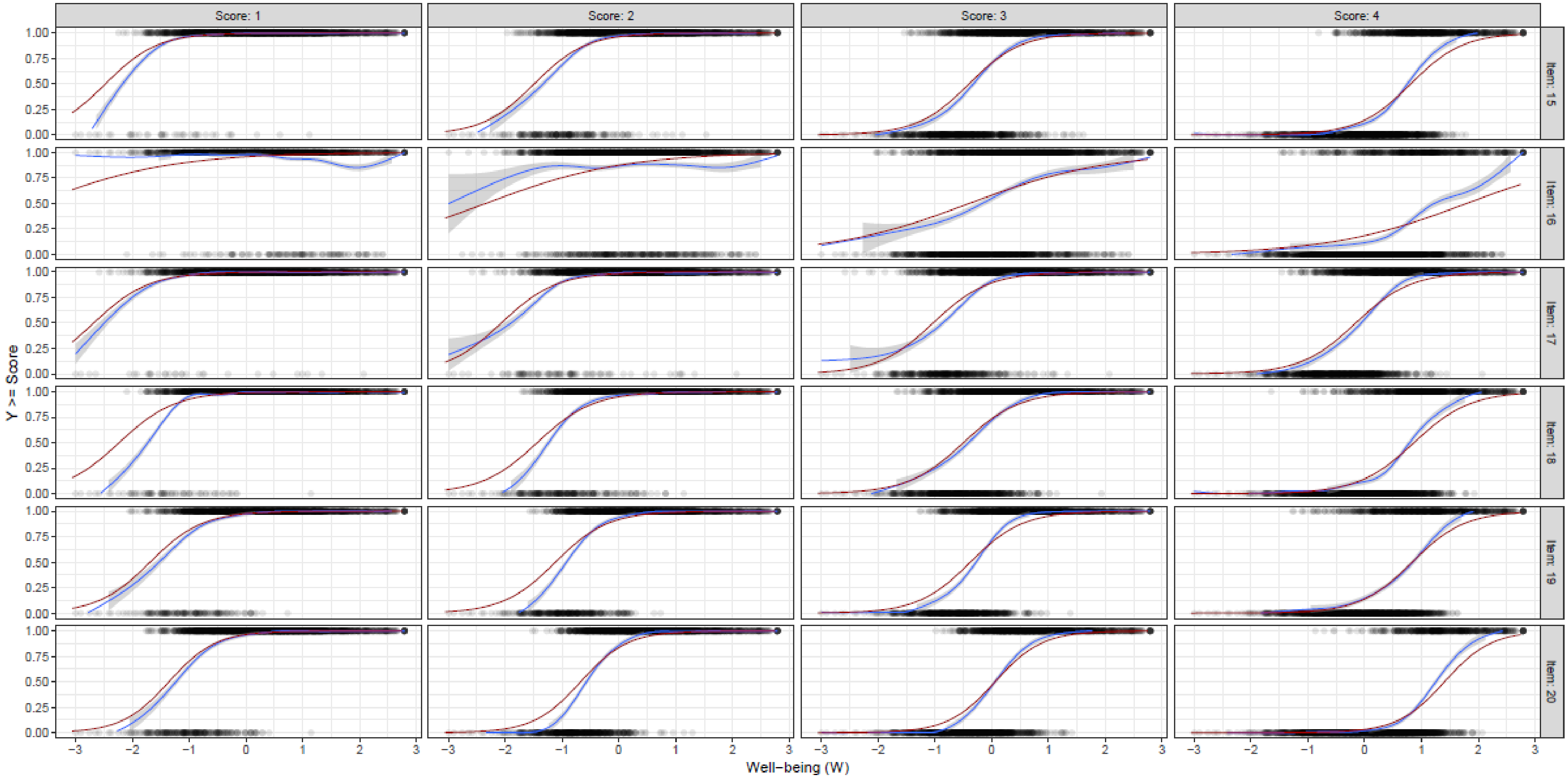

Figure S3 (continued): Diagnostic plots comparing the IRT model fit (red line) to the fit of generalized additive model (GAM) with cross-validated cubic spline as a smoothing function (blue line with 95% confidence interval in grey). Item numbers are as described in Table S1.

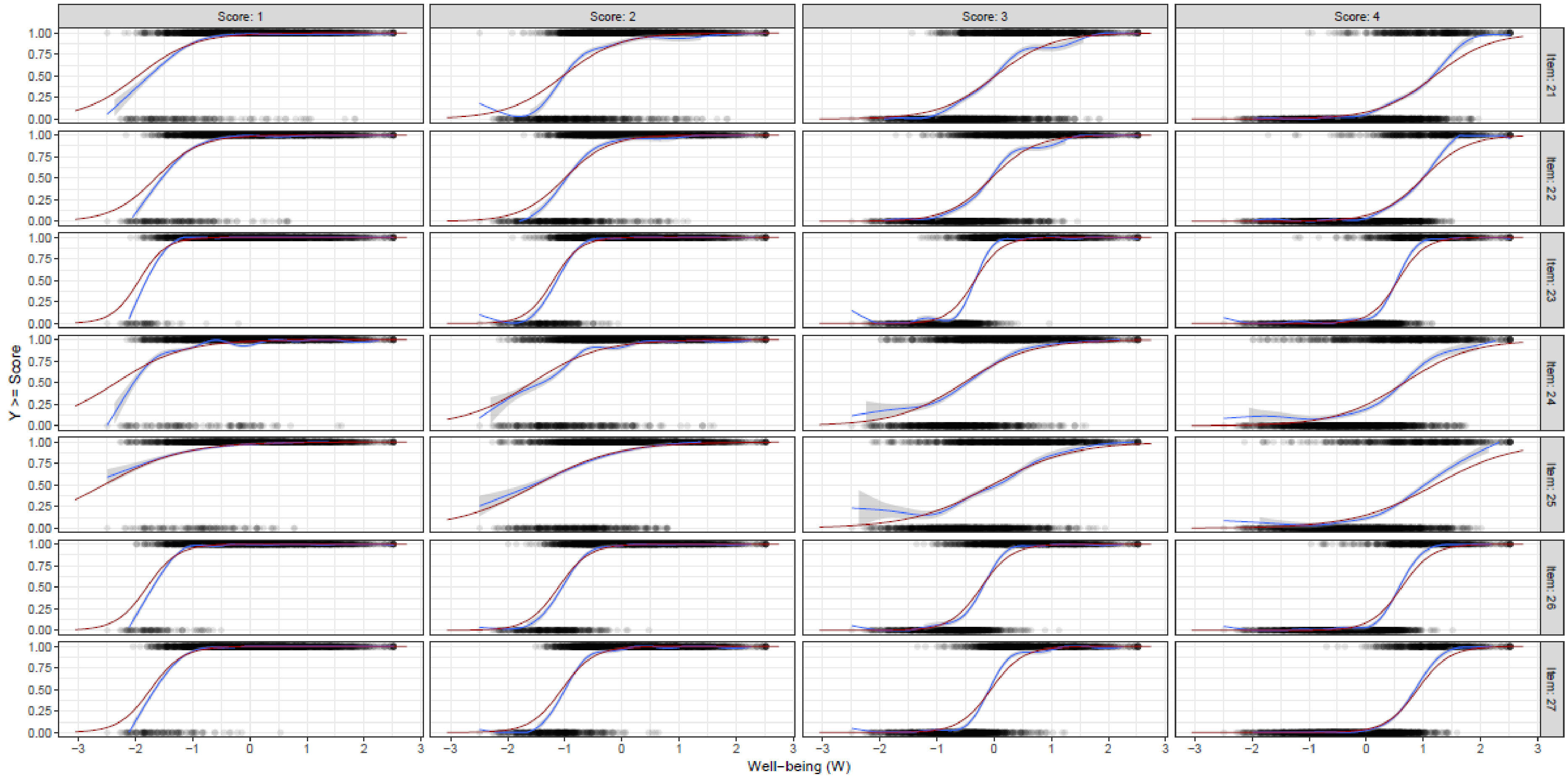

Figure S3 (continued): Diagnostic plots comparing the IRT model fit (red line) to the fit of generalized additive model (GAM) with cross-validated cubic spline as a smoothing function (blue line with 95% confidence interval in grey). Item numbers are as described in Table S1.

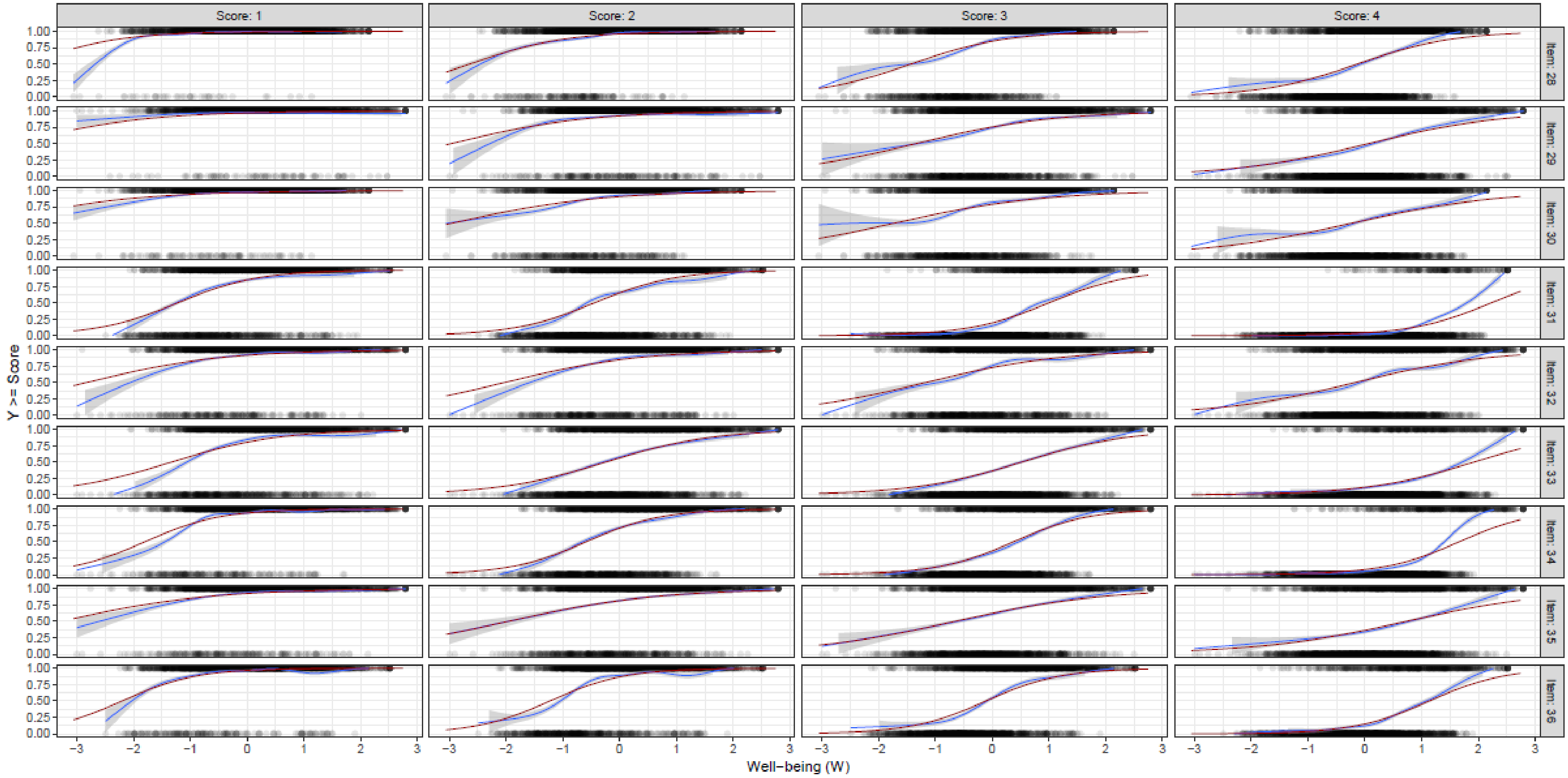

Figure S3 (continued): Diagnostic plots comparing the IRT model fit (red line) to the fit of generalized additive model (GAM) with cross-validated cubic spline as a smoothing function (blue line with 95% confidence interval in grey). Item numbers are as described in Table S1.

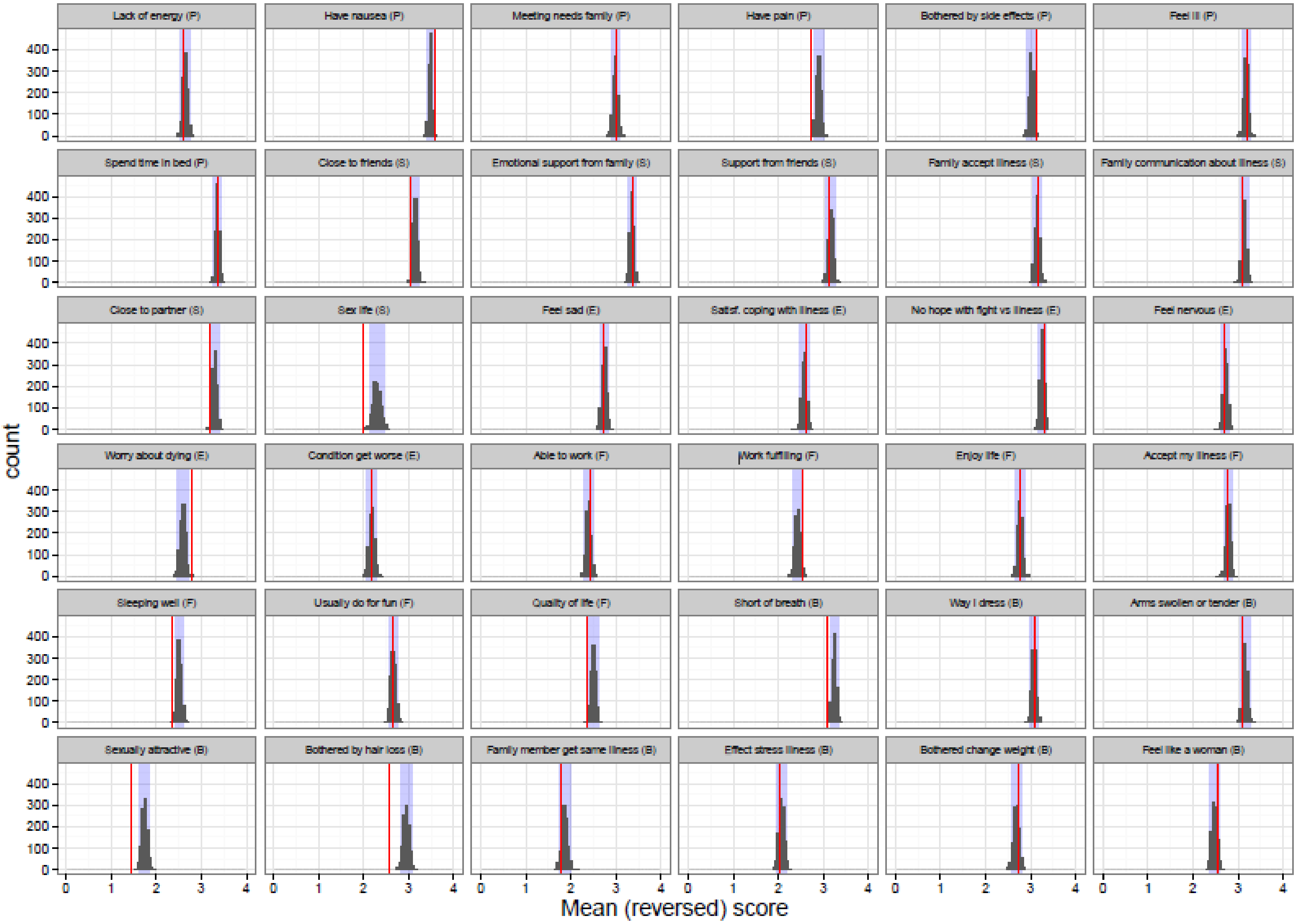

Figure S4: Mean scores for the original capecitabine-plus-lapatinib data set (red) compared to the mean score distribution in the simulated data sets obtained from the base IRT model (distribution in grey and 95% confidence interval in blue).

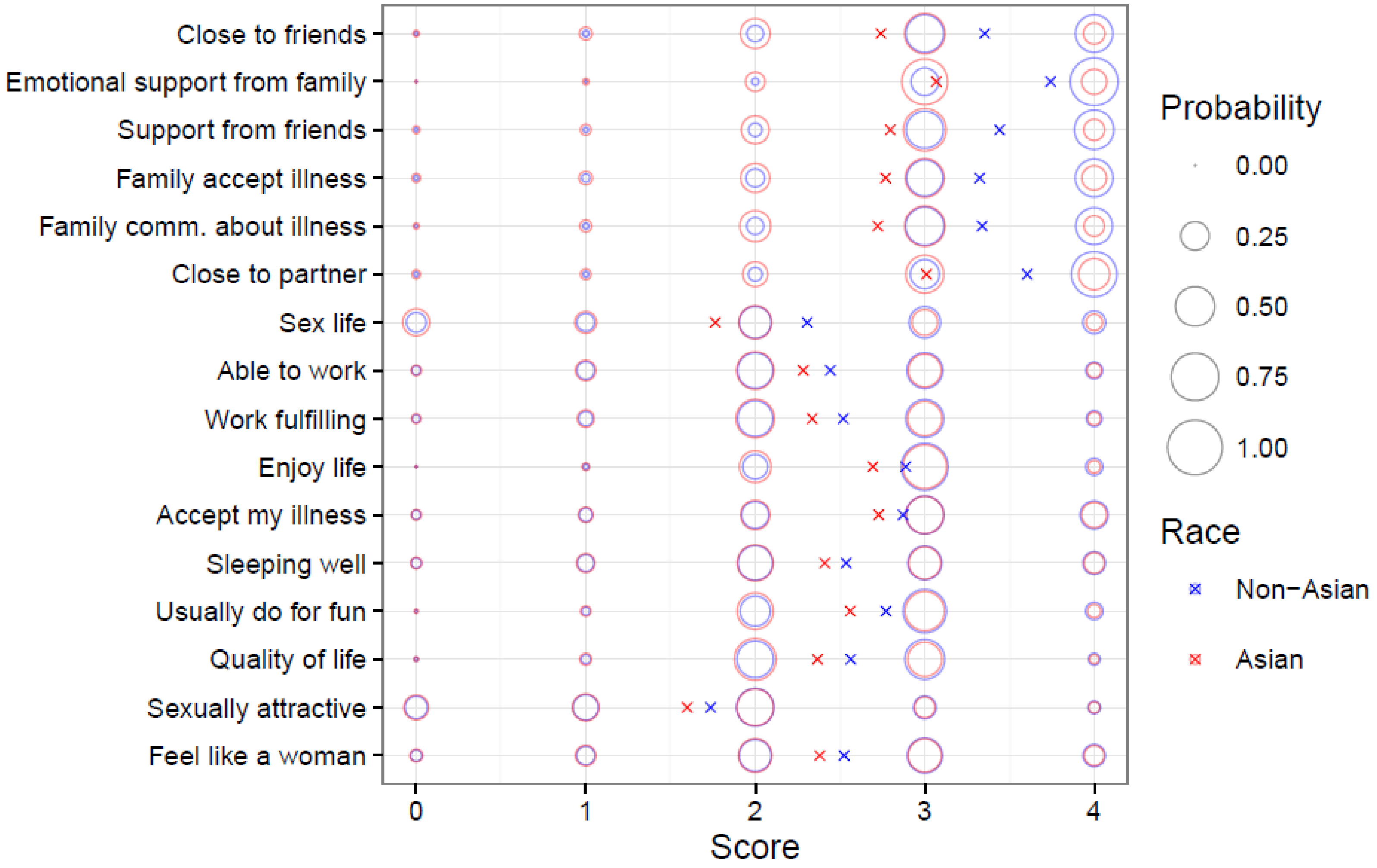

Figure S5: Schematic representation of the item score probabilities and expected score at baseline for a typical patient with baseline ECOG of 0, as predicted by the final longitudinal item-response theory model and differentiated by race. Circle surface areas are proportional to the score probability. Cross symbols represent expected scores, calculated as $\sum_{k=0}^{4} P(Y = k) \cdot k$. Scores for reverse items have been reverse-scaled (i.e. higher score indicate better outcome).

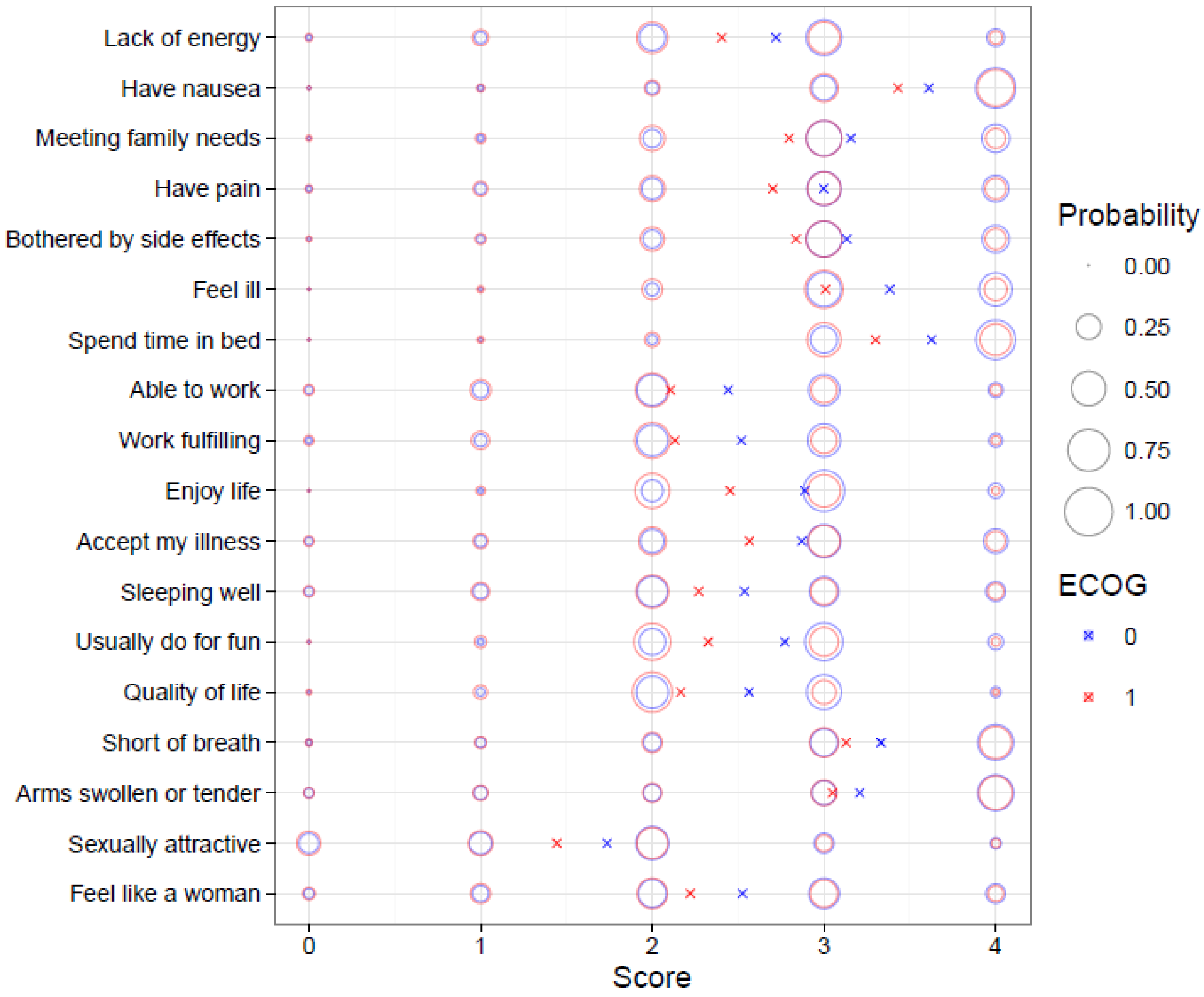

Figure S6: Schematic representation of the item score probabilities and expected score at baseline for a typical non-Asian patient, as predicted by the final longitudinal item-response theory model and differentiated by baseline ECOG. Circle surface areas are proportional to the score probability. Cross symbols represent expected scores, calculated as $\sum_{k=0}^{4} P(Y = k) \cdot k$. Scores for reverse items have been reverse-scaled (i.e. higher score indicate better outcome).
