# Supplementary material for: A Pharmacometric Analysis of Patient-Reported Outcomes in Breast Cancer Patients Through Item Response Theory
Source: Pharm Res. 2018 Apr 19;35(6):122. doi: 10.1007/s11095-018-2403-8 (PMC5908825; doi:10.1007/s11095-018-2403-8)
Supplement: Supplementary file 2 — (PDF 308 kb) [file 11095_2018_2403_MOESM2_ESM.pdf]

Supplementary material: NONMEM code and example data set

Example data set for the longitudinal IRT model

#ID	TRTACT	TIME	VAR	ODV	DV	MDV	BECOG	RACE2A
1	1	0	3	4	0	0	1	0
1	1	0	4	2	2	0	1	0
1	1	0	5	3	1	0	1	0
1	1	0	6	1	1	0	1	0
1	1	0	7	1	1	0	1	0
1	1	0	8	2	2	0	1	0
1	1	0	9	4	0	0	1	0
1	1	0	10	2	2	0	1	0
1	1	0	11	4	4	0	1	0
1	1	0	12	2	2	0	1	0
1	1	0	13	4	4	0	1	0
1	1	0	14	3	3	0	1	0
1	1	0	15	4	4	0	1	0
1	1	0	16	-99	-99	1	1	0
1	1	0	17	0	4	0	1	0
1	1	0	18	3	3	0	1	0
1	1	0	19	0	4	0	1	0
1	1	0	20	0	4	0	1	0
1	1	0	21	1	3	0	1	0
1	1	0	22	2	2	0	1	0
1	1	0	23	0	0	0	1	0
1	1	0	24	2	2	0	1	0
1	1	0	25	2	2	0	1	0
1	1	0	26	3	3	0	1	0
1	1	0	27	2	2	0	1	0
1	1	0	28	0	0	0	1	0
1	1	0	29	2	2	0	1	0
1	1	0	30	2	2	0	1	0
1	1	0	31	3	1	0	1	0
1	1	0	32	0	4	0	1	0
1	1	0	33	2	2	0	1	0
1	1	0	34	3	1	0	1	0
1	1	0	35	0	4	0	1	0
1	1	0	36	2	2	0	1	0
1	1	0	37	2	2	0	1	0
1	1	0	38	2	2	0	1	0
1	1	22	3	4	0	0	1	0
1	1	22	4	2	2	0	1	0
1	1	22	5	3	1	0	1	0
1	1	22	6	2	2	0	1	0
1	1	22	7	3	1	0	1	0
1	1	22	8	2	2	0	1	0
1	1	22	9	3	1	0	1	0
1	1	22	10	2	2	0	1	0
1	1	22	11	3	3	0	1	0
1	1	22	12	2	2	0	1	0
1	1	22	13	4	4	0	1	0
1	1	22	14	3	3	0	1	0
1	1	22	15	4	4	0	1	0
1	1	22	16	-99	-99	1	1	0
1	1	22	17	0	4	0	1	0
1	1	22	18	3	3	0	1	0
1	1	22	19	0	4	0	1	0
1	1	22	20	0	4	0	1	0
1	1	22	21	1	3	0	1	0
1	1	22	22	2	2	0	1	0
1	1	22	23	0	0	0	1	0
1	1	22	24	2	2	0	1	0
1	1	22	25	1	1	0	1	0
1	1	22	26	3	3	0	1	0
1	1	22	27	2	2	0	1	0
1	1	22	28	0	0	0	1	0
1	1	22	29	1	1	0	1	0
1	1	22	30	2	2	0	1	0
1	1	22	31	3	1	0	1	0
1	1	22	32	0	4	0	1	0
1	1	22	33	2	2	0	1	0
1	1	22	34	3	1	0	1	0
1	1	22	35	0	4	0	1	0
1	1	22	36	2	2	0	1	0
1	1	22	37	2	2	0	1	0
1	1	22	38	2	2	0	1	0

NONMEM code of the longitudinal IRT model

```
$SIZES      LTH=500 MAXIDS=50000 LNP4=10000
$PROBLEM    Longitudinal IRT model
$INPUT
ID          ; unique patient identifier
TRTACT      ; treatment arm (1=T-DM1, 2=capecitabine-plus-lapatinib)
TIME        ; time in days
VAR         ; variable: 3=item 1, 4=item 2, etc.
ODV         ; original dependent variable (score non-reversed)
DV          ; dependent variable (FACT-B (reversed) scores)
MDV         ; NONMEM missing data variable
BECOG       ; Baseline ECOG
RACE2A      ; Asian race

$DATA      example_dataset_FACTB.csv IGNORE=#
$PRED
;----------------------------Item parameters ----------------------------
; ~.~.~.~.~.~.~.~. Physical well-being ~.~.~.~.~.~.~.~.
IF(VAR.EQ.3) THEN                           ; GP1 - I have lack of energy
DISC = THETA(1)              ;DISC_GP1
DIF1 = THETA(2)              ;DIF1_GP1
DIF2 = THETA(3)              ;DIF2_GP1
DIF3 = THETA(4)              ;DIF3_GP1
DIF4 = THETA(5)              ;DIF4_GP1
ENDIF

IF(VAR.EQ.4) THEN                           ; GP2 - I have nausea
DISC = THETA(6)              ;DISC_GP2
DIF1 = THETA(7)              ;DIF1_GP2
DIF2 = THETA(8)              ;DIF2_GP2
DIF3 = THETA(9)              ;DIF3_GP2
DIF4 = THETA(10)             ;DIF4_GP2
ENDIF

IF(VAR.EQ.5) THEN                           ; GP3 - I have troubles meeting the needs of my
family
DISC = THETA(11)             ;DISC_GP3
DIF1 = THETA(12)             ;DIF1_GP3
DIF2 = THETA(13)             ;DIF2_GP3
DIF3 = THETA(14)             ;DIF3_GP3
DIF4 = THETA(15)             ;DIF4_GP3
ENDIF

IF(VAR.EQ.6) THEN                           ; GP4 - I have pain
DISC = THETA(16)              ;DISC_GP4
DIF1 = THETA(17)             ;DIF1_GP4
DIF2 = THETA(18)             ;DIF2_GP4
DIF3 = THETA(19)             ;DIF3_GP4
DIF4 = THETA(20)             ;DIF4_GP4
ENDIF

IF(VAR.EQ.7) THEN                           ; GP5 - I am bothered by side effects of treatment
DISC = THETA(21)              ;DISC_GP5
DIF1 = THETA(22)             ;DIF1_GP5
DIF2 = THETA(23)             ;DIF2_GP5
DIF3 = THETA(24)             ;DIF3_GP5
DIF4 = THETA(25)             ;DIF4_GP5
ENDIF

IF(VAR.EQ.8) THEN                           ; GP6 - I feel ill
DISC = THETA(26)              ;DISC_GP6
DIF1 = THETA(27)             ;DIF1_GP6
DIF2 = THETA(28)             ;DIF2_GP6
DIF3 = THETA(29)             ;DIF3_GP6
DIF4 = THETA(30)             ;DIF4_GP6
ENDIF

IF(VAR.EQ.9) THEN                           ; GP7 - I am forced to spend time in bed
```

```
DISC = THETA(31)              ;DISC_GP7
DIF1 = THETA(32)             ;DIF1_GP7
DIF2 = THETA(33)             ;DIF2_GP7
DIF3 = THETA(34)             ;DIF3_GP7
DIF4 = THETA(35)             ;DIF4_GP7
ENDIF

; ~.~.~.~.~.~.~.~. Social/Family well-being ~.~.~.~.~.~.~.~.
IF(VAR.EQ.10) THEN                          ; GS1 - I feel close to my friends
DISC = THETA(36)              ;DISC_GS1
DIF1 = THETA(37)             ;DIF1_GS1
DIF2 = THETA(38)             ;DIF2_GS1
DIF3 = THETA(39)             ;DIF3_GS1
DIF4 = THETA(40)             ;DIF4_GS1
ENDIF

IF(VAR.EQ.11) THEN                          ; GS2 - I get emotional support from my family
DISC = THETA(41)              ;DISC_GS2
DIF1 = THETA(42)             ;DIF1_GS2
DIF2 = THETA(43)             ;DIF2_GS2
DIF3 = THETA(44)             ;DIF3_GS2
DIF4 = THETA(45)             ;DIF4_GS2
ENDIF

IF(VAR.EQ.12) THEN                          ; GS3 - I get support from my friends
DISC = THETA(46)              ;DISC_GS3
DIF1 = THETA(47)             ;DIF1_GS3
DIF2 = THETA(48)             ;DIF2_GS3
DIF3 = THETA(49)             ;DIF3_GS3
DIF4 = THETA(50)             ;DIF4_GS3
ENDIF

IF(VAR.EQ.13) THEN                          ; GS4 - My family has accepted my illness
DISC = THETA(51)             ;DISC_GS4
DIF1 = THETA(52)             ;DIF1_GS4
DIF2 = THETA(53)             ;DIF2_GS4
DIF3 = THETA(54)             ;DIF3_GS4
DIF4 = THETA(55)             ;DIF4_GS4
ENDIF

IF(VAR.EQ.14) THEN                          ; GS5 - I am satisfied with family communication
about my illness
DISC = THETA(56)             ;DISC_GS5
DIF1 = THETA(57)             ;DIF1_GS5
DIF2 = THETA(58)             ;DIF2_GS5
DIF3 = THETA(59)             ;DIF3_GS5
DIF4 = THETA(60)             ;DIF4_GS5
ENDIF

IF(VAR.EQ.15) THEN                          ; GS6 - I feel close to my partner (or the person who
is my main support)
DISC = THETA(61)              ;DISC_GS6
DIF1 = THETA(62)             ;DIF1_GS6
DIF2 = THETA(63)             ;DIF2_GS6
DIF3 = THETA(64)             ;DIF3_GS6
DIF4 = THETA(65)             ;DIF4_GS6
ENDIF

IF(VAR.EQ.16) THEN                          ; GS7 - I am satisfied with my sex life
DISC = THETA(66)              ;DISC_GS7
DIF1 = THETA(67)             ;DIF1_GS7
DIF2 = THETA(68)             ;DIF2_GS7
DIF3 = THETA(69)             ;DIF3_GS7
DIF4 = THETA(70)             ;DIF4_GS7
ENDIF

; ~.~.~.~.~.~.~.~. Emotional well-being ~.~.~.~.~.~.~.~.
IF(VAR.EQ.17) THEN                          ; GE1 - I feel sad
DISC = THETA(71)              ;DISC_GE1
```

```
DIF1 = THETA(72)              ;DIF1_GE1
DIF2 = THETA(73)              ;DIF2_GE1
DIF3 = THETA(74)              ;DIF3_GE1
DIF4 = THETA(75)              ;DIF4_GE1
ENDIF

IF(VAR.EQ.18) THEN                          ; GE2 - I am satisfied with how I am coping with my
illness
DISC = THETA(76)               ;DISC_GE2
DIF1 = THETA(77)              ;DIF1_GE2
DIF2 = THETA(78)              ;DIF2_GE2
DIF3 = THETA(79)              ;DIF3_GE2
DIF4 = THETA(80)              ;DIF4_GE2
ENDIF

IF(VAR.EQ.19) THEN                          ; GE3 - I am losing hope in the fight against illness
DISC = THETA(81)               ;DISC_GE3
DIF1 = THETA(82)              ;DIF1_GE3
DIF2 = THETA(83)              ;DIF2_GE3
DIF3 = THETA(84)              ;DIF3_GE3
DIF4 = THETA(85)              ;DIF4_GE3
ENDIF

IF(VAR.EQ.20) THEN                          ; GE4 - I feel nervous
DISC = THETA(86)               ;DISC_GE4
DIF1 = THETA(87)              ;DIF1_GE4
DIF2 = THETA(88)              ;DIF2_GE4
DIF3 = THETA(89)              ;DIF3_GE4
DIF4 = THETA(90)              ;DIF4_GE4
ENDIF

IF(VAR.EQ.21) THEN                          ; GE5 - I worry about dying
DISC = THETA(91)               ;DISC_GE5
DIF1 = THETA(92)              ;DIF1_GE5
DIF2 = THETA(93)              ;DIF2_GE5
DIF3 = THETA(94)              ;DIF3_GE5
DIF4 = THETA(95)              ;DIF4_GE5
ENDIF

IF(VAR.EQ.22) THEN                         ; GE6 - I worry that my condition will get worse
DISC = THETA(96)               ;DISC_GE6
DIF1 = THETA(97)              ;DIF1_GE6
DIF2 = THETA(98)              ;DIF2_GE6
DIF3 = THETA(99)              ;DIF3_GE6
DIF4 = THETA(100)             ;DIF4_GE6
ENDIF

; ~.~.~.~.~.~.~.~. Functional well-being ~.~.~.~.~.~.~.~.
IF(VAR.EQ.23) THEN                          ; GF1 - I am able to work (include work at home)
DISC = THETA(101)              ;DISC_GF1
DIF1 = THETA(102)             ;DIF1_GF1
DIF2 = THETA(103)             ;DIF2_GF1
DIF3 = THETA(104)             ;DIF3_GF1
DIF4 = THETA(105)             ;DIF4_GF1
ENDIF

IF(VAR.EQ.24) THEN                          ; GF2 - My work (include work at home) is fullfilling
DISC = THETA(106)              ;DISC_GF2
DIF1 = THETA(107)             ;DIF1_GF2
DIF2 = THETA(108)             ;DIF2_GF2
DIF3 = THETA(109)             ;DIF3_GF2
DIF4 = THETA(110)             ;DIF4_GF2
ENDIF

IF(VAR.EQ.25) THEN                          ; GF3 - I am able to enjoy life
DISC = THETA(111)              ;DISC_GF3
DIF1 = THETA(112)             ;DIF1_GF3
DIF2 = THETA(113)             ;DIF2_GF3
DIF3 = THETA(114)             ;DIF3_GF3
```

```
DIF4 = THETA(115)              ;DIF4_GF3
ENDIF

IF(VAR.EQ.26) THEN                         ; GF4 - I have accepted my illness
DISC = THETA(116)              ;DISC_GF4
DIF1 = THETA(117)              ;DIF1_GF4
DIF2 = THETA(118)              ;DIF2_GF4
DIF3 = THETA(119)              ;DIF3_GF4
DIF4 = THETA(120)              ;DIF4_GF4
ENDIF

IF(VAR.EQ.27) THEN                         ; GF5 - I am sleeping well
DISC = THETA(121)               ;DISC_GF5
DIF1 = THETA(122)              ;DIF1_GF5
DIF2 = THETA(123)              ;DIF2_GF5
DIF3 = THETA(124)              ;DIF3_GF5
DIF4 = THETA(125)              ;DIF4_GF5
ENDIF

IF(VAR.EQ.28) THEN                         ; GF6 - I am enjoying the things I usually do for fun
DISC = THETA(126)               ;DISC_GF6
DIF1 = THETA(127)              ;DIF1_GF6
DIF2 = THETA(128)              ;DIF2_GF6
DIF3 = THETA(129)              ;DIF3_GF6
DIF4 = THETA(130)              ;DIF4_GF6
ENDIF

IF(VAR.EQ.29) THEN                         ; GF7 - I am content with the quality of my life
right now
DISC = THETA(131)               ;DISC_GF7
DIF1 = THETA(132)              ;DIF1_GF7
DIF2 = THETA(133)              ;DIF2_GF7
DIF3 = THETA(134)              ;DIF3_GF7
DIF4 = THETA(135)              ;DIF4_GF7
ENDIF

; ~.~.~.~.~.~.~.~. Breast cancer subscale ~.~.~.~.~.~.~.~.
IF(VAR.EQ.30) THEN                         ; B1 - I have been short of breath
DISC = THETA(136)               ;DISC_B1
DIF1 = THETA(137)              ;DIF1_B1
DIF2 = THETA(138)              ;DIF2_B1
DIF3 = THETA(139)              ;DIF3_B1
DIF4 = THETA(140)              ;DIF4_B1
ENDIF

IF(VAR.EQ.31) THEN                         ; B2 - I am self-conscious about the way I dress
DISC = THETA(141)               ;DISC_B2
DIF1 = THETA(142)              ;DIF1_B2
DIF2 = THETA(143)              ;DIF2_B2
DIF3 = THETA(144)              ;DIF3_B2
DIF4 = THETA(145)              ;DIF4_B2
ENDIF

IF(VAR.EQ.32) THEN                         ; B3 - One or both of my arms are swollen or tender
DISC = THETA(146)               ;DISC_B3
DIF1 = THETA(147)              ;DIF1_B3
DIF2 = THETA(148)              ;DIF2_B3
DIF3 = THETA(149)              ;DIF3_B3
DIF4 = THETA(150)              ;DIF4_B3
ENDIF

IF(VAR.EQ.33) THEN                         ; B4 - I feel sexually attractive
DISC = THETA(151)               ;DISC_B4
DIF1 = THETA(152)              ;DIF1_B4
DIF2 = THETA(153)              ;DIF2_B4
DIF3 = THETA(154)              ;DIF3_B4
DIF4 = THETA(155)              ;DIF4_B4
ENDIF
```

```
IF(VAR.EQ.34) THEN                          ; B5 - I am bothered by hair loss
DISC = THETA(156)              ;DISC_B5
DIF1 = THETA(157)             ;DIF1_B5
DIF2 = THETA(158)             ;DIF2_B5
DIF3 = THETA(159)             ;DIF3_B5
DIF4 = THETA(160)             ;DIF4_B5
ENDIF

IF(VAR.EQ.35) THEN                          ; B6 - I worry that other members of my family get
same illness
DISC = THETA(161)              ;DISC_B6
DIF1 = THETA(162)             ;DIF1_B6
DIF2 = THETA(163)             ;DIF2_B6
DIF3 = THETA(164)             ;DIF3_B6
DIF4 = THETA(165)             ;DIF4_B6
ENDIF

IF(VAR.EQ.36) THEN                          ; B7 - I worry about the effect of stress on my
illness
DISC = THETA(166)              ;DISC_B7
DIF1 = THETA(167)             ;DIF1_B7
DIF2 = THETA(168)             ;DIF2_B7
DIF3 = THETA(169)             ;DIF3_B7
DIF4 = THETA(170)             ;DIF4_B7
ENDIF

IF(VAR.EQ.37) THEN                          ; B8 - I am bothered by a change of weight
DISC = THETA(171)              ;DISC_B8
DIF1 = THETA(172)             ;DIF1_B8
DIF2 = THETA(173)             ;DIF2_B8
DIF3 = THETA(174)             ;DIF3_B8
DIF4 = THETA(175)             ;DIF4_B8
ENDIF

IF(VAR.EQ.38) THEN                          ; B9 - I am able to feel like a woman
DISC = THETA(176)              ;DISC_B9
DIF1 = THETA(177)             ;DIF1_B9
DIF2 = THETA(178)             ;DIF2_B9
DIF3 = THETA(179)             ;DIF3_B9
DIF4 = THETA(180)             ;DIF4_B9
ENDIF

;----------------------------- Baseline -----------------------------------------
; Define covariate relationships
IF(RACE2A.EQ.0) BASESRACE2A = 0  ; Most common
IF(RACE2A.EQ.1) BASESRACE2A = ( 0 + THETA(200))
IF(RACE2A.EQ.-99) BASESRACE2A = 0  ; Missing data

BASESCOV=BASESRACE2A

IF(BECOG.EQ.0) BASEPBECOG = 0  ; Most common
IF(BECOG.EQ.1) BASEPBECOG = ( 0 + THETA(197))
IF(BECOG.EQ.-99) BASEPBECOG = 0  ; Missing data

BASEPCOV=BASEPBECOG

IF(RACE2A.EQ.0) BASEFRACE2A = 0  ; Most common
IF(RACE2A.EQ.1) BASEFRACE2A = ( 0 + THETA(198))
IF(RACE2A.EQ.-99) BASEFRACE2A = 0  ; Missing data

IF(BECOG.EQ.0) BASEFBECOG = 0  ; Most common
IF(BECOG.EQ.1) BASEFBECOG = ( 0 + THETA(197))
IF(BECOG.EQ.-99) BASEFBECOG = 0  ; Missing data

BASEFCOV=BASEFBECOG+BASEFRACE2A

; Baseline W_0
TVBASEP = THETA(181)
```

```
TVBASEP = BASEPCOV+TVBASEP
TVBASES = THETA(182)

TVBASES = BASESCOV+TVBASES
TVBASEE = THETA(183)
TVBASEF = THETA(184)

TVBASEF = BASEFCOV+TVBASEF

BASEP   = TVBASEP + ETA(1)
BASES   = TVBASES + ETA(2)
BASEE   = TVBASEE + ETA(3)
BASEF   = TVBASEF + ETA(4)

;------------------------------ Time effect --------------------------------------
; Asymptote W_ss
IF(TRTACT.EQ.1) THEN   ; T-DM1
 TVPROP  = THETA(185)
 TVPROS  = THETA(186)
 TVPROE  = THETA(187)
 TVPROF  = THETA(188)
 ELSE                  ; Control
 TVPROP  = THETA(189)
 TVPROS  = THETA(190)
 TVPROE  = THETA(187)
 TVPROF  = THETA(192)
ENDIF

PROGP   = TVPROP + ETA(5) + ETA(6) ; Common IIV and ISV for both arms
PROGS   = TVPROS + ETA(5) + ETA(7)
PROGE   = TVPROE + ETA(5) + ETA(8)
PROGF   = TVPROF + ETA(5) + ETA(9)

; Half-life T_1/2
HLPROGP = THETA(193) *EXP(ETA(10)) ; Common TV and IIV for both arms
HLPROGS = THETA(194) *EXP(ETA(10))
HLPROGE = THETA(195) *EXP(ETA(10))
HLPROGF = THETA(196) *EXP(ETA(10))

;------------------------------Hidden variable model------------------------------
PWB = BASEP + PROGP*(1-EXP(-LOG(2)/HLPROGP*TIME))
SWB = BASES + PROGS*(1-EXP(-LOG(2)/HLPROGS*TIME))
EWB = BASEE + PROGE*(1-EXP(-LOG(2)/HLPROGE*TIME))
FWB = BASEF + PROGF*(1-EXP(-LOG(2)/HLPROGF*TIME))

;----------------------  model implementation grades 0 - 4 ----------------------
IF(VAR.GE.3.AND.VAR.LE.9)   PSI = PWB                ; Physical well-being
IF(VAR.GE.10.AND.VAR.LE.16) PSI = SWB                ; Social/Family well-being
IF(VAR.GE.17.AND.VAR.LE.22) PSI = EWB                ; Emotional well-being
IF(VAR.GE.23.AND.VAR.LE.29) PSI = FWB                ; Functional well-being

; -------- Reassign breast cancer subscale
IF(VAR.EQ.30) PSI = PWB                ; Short of breath
IF(VAR.EQ.31) PSI = EWB                ; way I dress
IF(VAR.EQ.32) PSI = PWB                ; arms swollen or tender
IF(VAR.EQ.33) PSI = FWB                ; sexually attractive
IF(VAR.GE.34.AND.VAR.LE.37) PSI = EWB  ; bothered by hair loss - family mb get same illness -
effect of stress - change of wt
IF(VAR.EQ.38) PSI = FWB                ; feel like a woman

; constrain DIFG5 >= DIFG4 >= DIFG3 >= DIFG2>= DIFG1
DIFG1 = DIF1
DIFG2 = DIFG1+DIF2
DIFG3 = DIFG2+DIF3
DIFG4 = DIFG3+DIF4
; DIFGx corresponds to b_j,x in the manuscript

; probability Y>=k, parameterized to avoid overflow
E1 = DISC*(PSI-DIFG1)
```

```
E2 = DISC*(PSI-DIFG2)
E3 = DISC*(PSI-DIFG3)
E4 = DISC*(PSI-DIFG4)

PGE1 = EXP(E1)/(1+EXP(E1))
PGE2 = EXP(E2)/(1+EXP(E2))
PGE3 = EXP(E3)/(1+EXP(E3))
PGE4 = EXP(E4)/(1+EXP(E4))

IF(E1.GT.0) PGE1 = 1/(1+EXP(-E1))
IF(E2.GT.0) PGE2 = 1/(1+EXP(-E2))
IF(E3.GT.0) PGE3 = 1/(1+EXP(-E3))
IF(E4.GT.0) PGE4 = 1/(1+EXP(-E4))

; probability Y==k
P0 = 1-PGE1
P1 = PGE1-PGE2
P2 = PGE2-PGE3
P3 = PGE3-PGE4
P4 = PGE4

IF(DV.EQ.0) P = P0
IF(DV.EQ.1) P = P1
IF(DV.EQ.2) P = P2
IF(DV.EQ.3) P = P3
IF(DV.EQ.4) P = P4

;------------------------Response probability prediction-------------------------
IF(P.LT.1E-16)     P = 1E-16     ; protection for P->0
IF(P.GT.(1-1E-16)) P = 1-1E-16   ; protection for P->1
Y = -2*LOG(P)

;--------------------------------Simulation code---------------------------------
REP=IREP
IF(ICALL.EQ.4) THEN
CALL RANDOM (2,R)        ; Call random variable from a uniform distribution
SDV = 0                  ; Simulated DV
IF(R.LT.PGE1) SDV = 1
IF(R.LT.PGE2) SDV = 2
IF(R.LT.PGE3) SDV = 3
IF(R.LT.PGE4) SDV = 4
DV = SDV
ENDIF

$ESTIMATION MAXEVAL=0 METHOD=COND LAPLACE -2LL PRINT=1

$THETA  (0,2.06967) FIX ; 1        DISC_GP1
 -2.32367 FIX ; 2        DIF1_GP1
 (0,0.973293,1000000) FIX ; 3 DIF2_GP1
 (0,1.02383,1000000) FIX ; 4  DIF3_GP1
 (0,1.27388,1000000) FIX ; 5  DIF4_GP1
 (0,1.46945) FIX ; 6  DISC_GP2
 -3.99677 FIX ; 7      DIF1_GP2
 (0,1.02473,1000000) FIX ; 8  DIF2_GP2
 (0,1.15742,1000000) FIX ; 9  DIF3_GP2
 (0,1.26797,1000000) FIX ; 10 DIF4_GP2
 (0,2.55835) FIX ; 11  DISC_GP3
 -2.25456 FIX ; 12     DIF1_GP3
 (0,0.68862,1000000) FIX ; 13 DIF2_GP3
 (0,0.876377,1000000) FIX ; 14       DIF3_GP3
 (0,0.977248,1000000) FIX ; 15       DIF4_GP3
 (0,1.74575) FIX ; 16  DISC_GP4
 -2.7107 FIX ; 17      DIF1_GP4
 (0,1.0448,1000000) FIX ; 18  DIF2_GP4
 (0,0.964461,1000000) FIX ; 19       DIF3_GP4
 (0,1.22583,1000000) FIX ; 20 DIF4_GP4
 (0,2.06659) FIX ; 21  DISC_GP5
 -2.69692 FIX ; 22     DIF1_GP5
 (0,0.878561,1000000) FIX ; 23       DIF2_GP5
```

```
(0,0.991375,1000000) FIX ; 24       DIF3_GP5
(0,1.21088,1000000) FIX ; 25 DIF4_GP5
(0,3.14735) FIX ; 26  DISC_GP6
-2.46072 FIX ; 27    DIF1_GP6
(0,0.736756,1000000) FIX ; 28       DIF2_GP6
(0,0.870586,1000000) FIX ; 29       DIF3_GP6
(0,0.914412,1000000) FIX ; 30       DIF4_GP6
(0,2.83468) FIX ; 31  DISC_GP7
-2.63326 FIX ; 32    DIF1_GP7
(0,0.764731,1000000) FIX ; 33       DIF2_GP7
(0,0.708716,1000000) FIX ; 34       DIF3_GP7
(0,0.91286,1000000) FIX ; 35 DIF4_GP7
(0,3.0398) FIX ; 36   DISC_GS1
-2.17664 FIX ; 37    DIF1_GS1
(0,0.629604,1000000) FIX ; 38       DIF2_GS1
(0,0.718636,1000000) FIX ; 39       DIF3_GS1
(0,0.831105,1000000) FIX ; 40       DIF4_GS1
(0,4.67679) FIX ; 41  DISC_GS2
-2.10385 FIX ; 42    DIF1_GS2
(0,0.477812,1000000) FIX ; 43       DIF2_GS2
(0,0.593268,1000000) FIX ; 44       DIF3_GS2
(0,0.735385,1000000) FIX ; 45       DIF4_GS2
(0,3.54032) FIX ; 46  DISC_GS3
-1.85062 FIX ; 47    DIF1_GS3
(0,0.357667,1000000) FIX ; 48       DIF2_GS3
(0,0.623387,1000000) FIX ; 49       DIF3_GS3
(0,0.80675,1000000) FIX ; 50 DIF4_GS3
(0,2.44827) FIX ; 51  DIF4_GS4
-2.24657 FIX ; 52    DIF1_GS4
(0,0.566298,1000000) FIX ; 53       DIF2_GS4
(0,0.792694,1000000) FIX ; 54       DIF3_GS4
(0,0.890613,1000000) FIX ; 55       DIF4_GS4
(0,3.13366) FIX ; 56  DISC_GS5
-2.17512 FIX ; 57    DIF1_GS5
(0,0.596025,1000000) FIX ; 58       DIF2_GS5
(0,0.780144,1000000) FIX ; 59       DIF3_GS5
(0,0.821042,1000000) FIX ; 60       DIF4_GS5
(0,2.87691) FIX ; 61  DISC_GS6
-2.0057 FIX ; 62     DIF1_GS6
(0,0.350071,1000000) FIX ; 63       DIF2_GS6
(0,0.639858,1000000) FIX ; 64       DIF3_GS6
(0,0.698017,1000000) FIX ; 65       DIF4_GS6
(0,1.48866) FIX ; 66  DISC_GS7
-1.42423 FIX ; 67    DIF1_GS7
(0,0.465285,1000000) FIX ; 68       DIF2_GS7
(0,0.979391,1000000) FIX ; 69       DIF3_GS7
(0,1.04301,1000000) FIX ; 70 DIF4_GS7
(0,2.19028) FIX ; 71  DISC_GE1
-2.47112 FIX ; 72    DIF1_GE1
(0,0.982771,1000000) FIX ; 73       DIF2_GE1
(0,1.09003,1000000) FIX ; 74 DIF3_GE1
(0,1.21093,1000000) FIX ; 75 DIF4_GE1
(0,0.829776) FIX ; 76 DISC_GE2
-3.72614 FIX ; 77    DIF1_GE2
(0,1.38475,1000000) FIX ; 78 DIF2_GE2
(0,1.94605,1000000) FIX ; 79 DIF3_GE2
(0,2.19051,1000000) FIX ; 80 DIF4_GE2
(0,2.06367) FIX ; 81  DISC_GE3
-2.67833 FIX ; 82    DIF1_GE3
(0,0.610841,1000000) FIX ; 83       DIF2_GE3
(0,1.03572,1000000) FIX ; 84 DIF3_GE3
(0,0.875755,1000000) FIX ; 85       DIF4_GE3
(0,2.04486) FIX ; 86  DISC_GE4
-2.22963 FIX ; 87    DIF1_GE4
(0,0.793616,1000000) FIX ; 88       DIF2_GE4
(0,0.938793,1000000) FIX ; 89       DIF3_GE4
(0,1.36667,1000000) FIX ; 90 DIF4_GE4
(0,2.18015) FIX ; 91  DISC_GE5
-1.68771 FIX ; 92    DIF1_GE5
```

```
(0,0.571225,1000000) FIX ; 93        DIF2_GE5
(0,0.739694,1000000) FIX ; 94        DIF3_GE5
(0,1.21331,1000000) FIX ; 95 DIF4_GE5
(0,2.42857) FIX ; 96  DISC_GE6
-1.35576 FIX ; 97     DIF1_GE6
(0,0.683,1000000) FIX ; 98   DIF2_GE6
(0,0.731477,1000000) FIX ; 99        DIF3_GE6
(0,1.34569,1000000) FIX ; 100        DIF4_GE6
(0,2.03488) FIX ; 101 DISC_GF1
-1.95685 FIX ; 102    DIF1_GF1
(0,0.925233,1000000) FIX ; 103       DIF2_GF1
(0,1.05317,1000000) FIX ; 104        DIF3_GF1
(0,1.17342,1000000) FIX ; 105        DIF4_GF1
(0,2.61787) FIX ; 106 DISC_GF2
-1.64736 FIX ; 107    DIF1_GF2
(0,0.647825,1000000) FIX ; 108       DIF2_GF2
(0,0.952437,1000000) FIX ; 109       DIF3_GF2
(0,1.03989,1000000) FIX ; 110        DIF4_GF2
(0,4.27106) FIX ; 111 DISC_GF3
-1.93658 FIX ; 112    DIF1_GF3
(0,0.735048,1000000) FIX ; 113       DIF2_GF3
(0,0.864236,1000000) FIX ; 114       DIF3_GF3
(0,0.892655,1000000) FIX ; 115       DIF4_GF3
(0,1.68813) FIX ; 116 DISC_GF4
-2.33858 FIX ; 117    DIF1_GF4
(0,0.762524,1000000) FIX ; 118       DIF2_GF4
(0,1.0446,1000000) FIX ; 119 DIF3_GF4
(0,1.1959,1000000) FIX ; 120 DIF4_GF4
(0,1.43132) FIX ; 121 DISC_GF5
-2.55648 FIX ; 122    DIF1_GF5
(0,1.05813,1000000) FIX ; 123        DIF2_GF5
(0,1.46053,1000000) FIX ; 124        DIF3_GF5
(0,1.23445,1000000) FIX ; 125        DIF4_GF5
(0,3.75996) FIX ; 126 DISC_GF6
-1.79901 FIX ; 127    DIF1_GF6
(0,0.689372,1000000) FIX ; 128       DIF2_GF6
(0,0.885276,1000000) FIX ; 129       DIF3_GF6
(0,0.844313,1000000) FIX ; 130       DIF4_GF6
(0,3.63157) FIX ; 131 DISC_GF7
-1.73504 FIX ; 132    DIF1_GF7
(0,0.683668,1000000) FIX ; 133       DIF2_GF7
(0,0.996186,1000000) FIX ; 134       DIF3_GF7
(0,0.952683,1000000) FIX ; 135       DIF4_GF7
(0,1.19272) FIX ; 136 DISC_B1
-3.90982 FIX ; 137    DIF1_B1
(0,1.27754,1000000) FIX ; 138        DIF2_B1
(0,1.20796,1000000) FIX ; 139        DIF3_B1
(0,1.29881,1000000) FIX ; 140        DIF4_B1
(0,0.834601) FIX ; 141       DISC_B2
-4.13157 FIX ; 142    DIF1_B2
(0,1.17503,1000000) FIX ; 143        DIF2_B2
(0,1.62091,1000000) FIX ; 144        DIF3_B2
(0,1.40491,1000000) FIX ; 145        DIF4_B2
(0,0.765407) FIX ; 146       DISC_B3
-4.59083 FIX ; 147    DIF1_B3
(0,1.64436,1000000) FIX ; 148        DIF2_B3
(0,1.22619,1000000) FIX ; 149        DIF3_B3
(0,1.49325,1000000) FIX ; 150        DIF4_B3
(0,1.41193) FIX ; 151 DISC_B4
-1.2194 FIX ; 152     DIF1_B4
(0,0.776052,1000000) FIX ; 153       DIF2_B4
(0,1.44336,1000000) FIX ; 154        DIF3_B4
(0,1.22818,1000000) FIX ; 155        DIF4_B4
(0,0.839776) FIX ; 156       DISC_B5
-2.81542 FIX ; 157    DIF1_B5
(0,0.796335,1000000) FIX ; 158       DIF2_B5
(0,0.853956,1000000) FIX ; 159       DIF3_B5
(0,1.00399,1000000) FIX ; 160        DIF4_B5
(0,1.05692) FIX ; 161 DISC_B6
```

```
 -1.32897 FIX ; 162    DIF1_B6
 (0,1.08275,1000000) FIX ; 163       DIF2_B6
 (0,0.766046,1000000) FIX ; 164      DIF3_B6
 (0,1.40185,1000000) FIX ; 165       DIF4_B6
 (0,1.50985) FIX ; 166 DISC_B7
 -1.79547 FIX ; 167    DIF1_B7
 (0,1.20925,1000000) FIX ; 168       DIF2_B7
 (0,0.963077,1000000) FIX ; 169      DIF3_B7
 (0,1.28856,1000000) FIX ; 170       DIF4_B7
 (0,0.763562) FIX ; 171       DISC_B8
 -3.27534 FIX ; 172    DIF1_B8
 (0,1.31906,1000000) FIX ; 173       DIF2_B8
 (0,1.30604,1000000) FIX ; 174       DIF3_B8
 (0,1.38463,1000000) FIX ; 175       DIF4_B8
 (0,1.52447) FIX ; 176 DISC_B9
 -2.20007 FIX ; 177    DIF1_B9
 (0,0.975209,1000000) FIX ; 178      DIF2_B9
 (0,1.1158,1000000) FIX ; 179 DIF3_B9
 (0,1.24656,1000000) FIX ; 180       DIF4_B9
 0 FIX ; 181 BASE_PWB
 -0.0901136 ; 182 BASE_SWB
 0 FIX ; 183 BASE_EWB
 0 FIX ; 184 BASE_FWB
 0 FIX ; 185 PROGP TDM1
 -0.243662 ; 185 PROG_SWB
 0.294583 ; 186 PROG_EWB
 0 FIX ; 188 PROGF TDM1
 -0.250895 ; 189 PROG_PWB REF
 -0.13742 ; 190 PROG_SWB REF
 0 FIX ; 191 PROGE CONTROL
 0 FIX ; 192 PROGF CONTROL
 (0,30.6837) ; 193 HLPROGP
 (0,116.559) ; 194 HLPROGS
 (0,35.0815) ; 195 HLPROGE
 (0,48.9207) ; 196 HLPROGF
$THETA  (-20,-0.268364,20) ; 197 BASEFPBECOG1
$THETA  (-20,-0.181025,20) ; 198 BASEFRACE2A1
$THETA  0 FIX
$THETA  (-20,-0.441136,20) ; 200 BASESRACE2A1
$OMEGA  BLOCK(5)
 0.574243  ; OM_BASE_PWB
 0.230733 0.475783  ; OM_BASE_SWB
 0.483043 0.282686 0.65536  ; OM_BASE_EWB
 0.479774 0.418151 0.441594 0.618078  ; OM_BASE_FWB
 -0.146212 -0.0939033 -0.136536 -0.138096 0.342357  ; OM_PROG_IIV ALL
$OMEGA  BLOCK(1)
 0.0267419  ; OM_PROG_ISV TDM1
$OMEGA  BLOCK(1) SAME
$OMEGA  BLOCK(1) SAME
$OMEGA  BLOCK(1) SAME
$OMEGA  1.45579  ; OM_HLPROGP TDM1
```
